# A Global Census of Fission Yeast Deubiquitinating Enzyme Localization and Interaction Networks Reveals Distinct Compartmentalization Profiles and Overlapping Functions in Endocytosis and Polarity

**DOI:** 10.1371/journal.pbio.1000471

**Published:** 2010-09-07

**Authors:** Ilektra Kouranti, Janel R. McLean, Anna Feoktistova, Ping Liang, Alyssa E. Johnson, Rachel H. Roberts-Galbraith, Kathleen L. Gould

**Affiliations:** 1 Department of Cell and Developmental Biology, Vanderbilt University School of Medicine, Nashville, Tennessee, United States of America; 2 Howard Hughes Medical Institute, Vanderbilt University School of Medicine, Nashville, Tennessee, United States of America; University of California San Francisco/Howard Hughes Medical Institute, United States of America

## Abstract

Ubiquitination and deubiquitination are reciprocal processes that tune protein stability, function, and/or localization. The removal of ubiquitin and remodeling of ubiquitin chains is catalyzed by deubiquitinating enzymes (DUBs), which are cysteine proteases or metalloproteases. Although ubiquitination has been extensively studied for decades, the complexity of cellular roles for deubiquitinating enzymes has only recently been explored, and there are still several gaps in our understanding of when, where, and how these enzymes function to modulate the fate of polypeptides. To address these questions we performed a systematic analysis of the 20 *Schizosaccharomyces pombe* DUBs using confocal microscopy, proteomics, and enzymatic activity assays. Our results reveal that *S. pombe* DUBs are present in almost all cell compartments, and the majority are part of stable protein complexes essential for their function. Interestingly, DUB partners identified by our study include the homolog of a putative tumor suppressor gene not previously linked to the ubiquitin pathway, and two conserved tryptophan-aspartate (WD) repeat proteins that regulate Ubp9, a DUB that we show participates in endocytosis, actin dynamics, and cell polarity. In order to understand how DUB activity affects these processes we constructed multiple DUB mutants and find that a quintuple deletion of *ubp4 ubp5 ubp9 ubp15 sst2/amsh* displays severe growth, polarity, and endocytosis defects. This mutant allowed the identification of two common substrates for five cytoplasmic DUBs. Through these studies, a common regulatory theme emerged in which DUB localization and/or activity is modulated by interacting partners. Despite apparently distinct cytoplasmic localization patterns, several DUBs cooperate in regulating endocytosis and cell polarity. These studies provide a framework for dissecting DUB signaling pathways in *S. pombe* and may shed light on DUB functions in metazoans.

## Abstract 2

Proteomic, localization, and enzymatic activity screens in fission yeast reveal how deubiquitinating enzyme localization and function are tuned.

## Author Summary

The post-translational modification of proteins by conjugation of monomers or chains of ubiquitin is a regulatory mechanism for tuning protein stability, localization and function. Given these vital functions, ubiquitination has to be highly regulated so that protein degradation and cell signaling are controlled in space and time. Although the ubiquitin-conjugation machinery has been thoroughly studied, there are still several gaps in our understanding of when, where and how ubiquitin is removed by deubiquitinating enzymes (DUBs). To address these questions we performed a systematic analysis of the 20 DUBs in the fission yeast *Schizosaccharomyces pombe* using confocal microscopy, proteomics and enzymatic activity assays. We first showed that *S. pombe* DUBs are present in almost all cell compartments and that the majority are part of stable protein complexes essential for their function. Then, we constructed strains mutant for a number of the DUBs involved in the newly identified protein complexes and showed that five cytoplasmic DUBs have redundant roles in controlling endocytosis and cell polarity. We postulate that regulatory networks identified in our study might be conserved and hence shed light on DUB function in metazoans.

## Introduction

Posttranslational modifications govern protein function by modulating their structure, localization, dynamics, and/or stability. Ubiquitination of substrate proteins induces an array of specific responses depending on the extent and architecture of the modification. Proteins can be modified by addition of a single ubiquitin on a single site (monoubiquitination) or multiple sites (multiple monoubiquitination) or by polymerization of ubiquitin monomers into chains of specific linkages (polyubiquitination) [1]. Specific ubiquitin configurations elicit unique cellular responses and affect essential processes including protein degradation, DNA repair, chromatin remodeling, endocytosis, and cell cycle regulation [1],[2]. Due to the vital roles of ubiquitination, this process is highly regulated and requires a cascade of three enzymes, culminating in a substrate- and site-specific modification [2]. Likewise, cleavage of ubiquitin moieties or chains by deubiquitinating enzymes (DUBs) must be tightly regulated in space and time [3].

DUBs are highly conserved cysteine proteases or metalloproteases that can be classified based on their catalytic domain structure: ubiquitin C-terminal hydrolases (UCHs), ubiquitin-specific proteases (USPs), ovarian tumor proteases (OTUs), Machado-Joseph disease proteases, and JAB1/MPN/Mov34 metalloenzymes (JAMMs) [4]. The diversity of DUB catalytic core and domain structures, as well as their number (approximately 95 DUBs encoded by the human genome), reflects their involvement in multiple essential roles including (1) processing of ubiquitin precursor proteins, (2) recycling of ubiquitin trapped in modified, inactivatable forms, (3) cleavage of ubiquitin from target proteins, and (4) regeneration of monoubiquitin from free polyubiquitin chains [3]–[5].

Specific functions of several DUBs have been elucidated. A trio of DUBs (Rpn11/PSMD14, Uch2/UCHL5, and Ubp6/USP14) act at the proteasome to remodel or remove ubiquitin chains prior to substrate degradation [6]–[10]. Other DUBs play roles in transcriptional regulation (Ubp8p/USP22), downregulation of the NFκB pathway (CYLD), DNA repair (USP1), or membrane trafficking between the endoplasmic reticulum (ER) and the Golgi complex (Ubp3p) [11]–[16]. Although a role for DUBs in several pathways has been defined, their enzymatic targets and modes of regulation remain largely unknown [17]. A recent proteomic study of human DUBs assigned potential roles to previously uncharacterized DUBs by placing them in putative cellular contexts defined mainly by the nature of their interactors [18]. However, despite such efforts to link various DUBs to different cellular functions in several organisms, there are still significant gaps in our understanding of the action and regulation of these enzymes.

**Figure 1:**
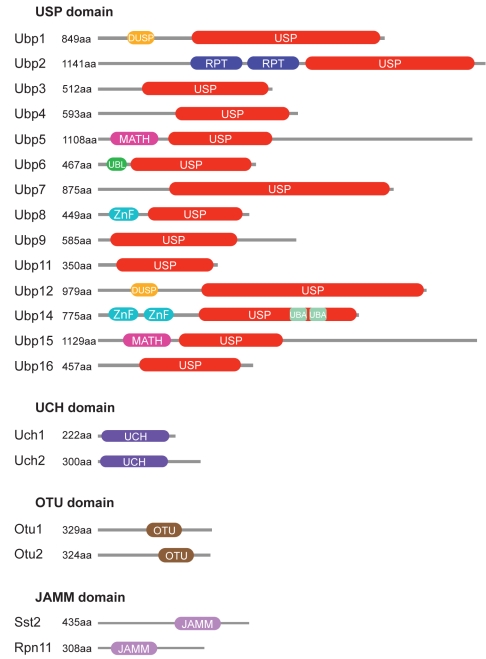
Inventory and domain architecture of *S. pombe* DUBs. *S. pombe* DUBs belong to four subfamilies (USP, UCH, OTU, and JAMM). USP, UCH, and OTU domain DUBs are cysteine proteases, JAMM domain DUBs are metalloproteases. We retrieved domain architectures for each DUB using the SMART and Pfam databases. The following domains were found: DUSP (domain in ubiquitin-specific proteases), MATH (meprin and TRAF homology), UBL (ubiquitin-like), ZnF (ubiquitin carboxyl-terminal hydrolase-like zinc finger), UBA (Ubiquitin-associated). RPT, internal repeats.

In this study, we characterize the entire family of DUBs in the fission yeast *Schizosaccharomyces pombe.* In contrast to mammalian cells, the *S. pombe* genome encodes only 20 putative DUBs belonging to four of the five DUB subfamilies (UCH, USP, OTU, and JAMM; Figure 1). A handful of other proteins in the *S. pombe* genome encode DUB domains (Ubp10, Ubp13, Rpn8, Csn5, Cwf6, eIF3f, and eIF3h), but they are either lacking the full complement of catalytic residues necessary for protease function (Figure S1) or, in the case of the signalosome component Csn5, have activity towards other ubiquitin-like proteins and have been excluded from our consideration [4],[19],[20]. All *S. pombe* DUBs are nonessential for viability, except for one of the proteasomal DUBs, Rpn11 [21]–[25]. We chose to study the *S. pombe* DUB family because of the limited number of DUBs encoded by this genome, the conservation of catalytic core structures and some non-catalytic domain modules (Figure 1) [4], and the genetic tractability of *S. pombe,* which allows endogenous gene tagging and simple genetic manipulation. These attributes confer a significant advantage for a genome-wide study and the potential to comprehensively assign DUB activities to functional networks.

We took a multifaceted approach to investigate *S. pombe* DUBs, combining the determination of endogenous localizations, evaluation of their in vitro activity, and proteomic analysis of protein interactions. To our knowledge, this work provides the first systematic localization study of a complete DUB family and reveals that *S. pombe* DUBs are present in nearly every cellular compartment. Moreover, our proteomic approach identified stable protein–protein interactions for over 55% of the *S. pombe* DUBs. By means of subcellular localization studies and activity assays we show how three uncharacterized DUBs are regulated by non-catalytic partners, including a potential interactor for human USP7/HAUSP, which controls the tumor suppressor p53 [26],[27]. We also found that a conserved DUB complex participates in endocytosis, actin organization, and cell polarity and that these cellular functions are shared by at least five different DUBs. The powerful combination of experimental approaches utilized in this study reveals new examples of regulation for this important protein family.

## Results

### Subcellular Localization of the *S. pombe* DUB Family

Only a few *S. pombe* DUBs have been studied in detail, in particular those associated with the 26S proteasome or the COP9 signalosome [21]–[23],[28],[29]. With sparse information available for *S. pombe* DUBs, we reasoned that the localization of these proteins would be a first step in placing each DUB into a functional category.

**Figure 2:**
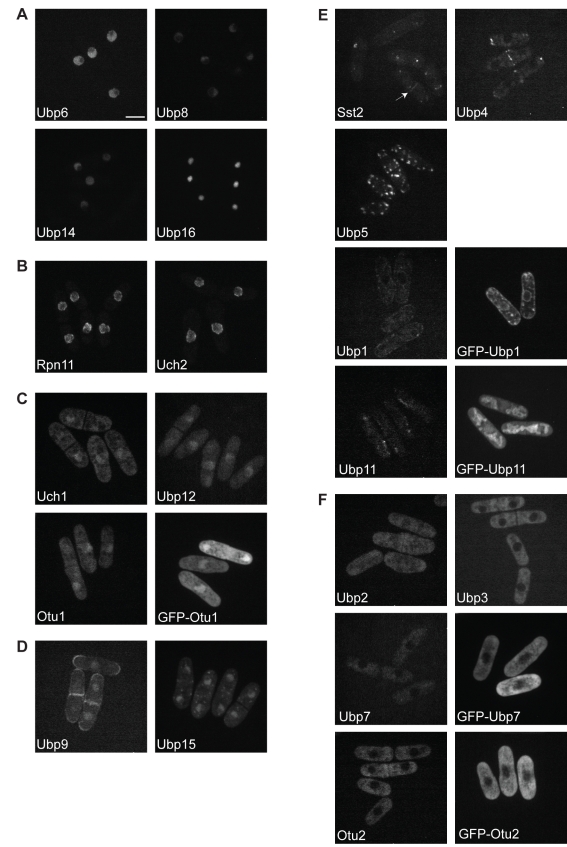
Localization of *S. pombe* DUBs. Cells producing DUBs endogenously tagged at their C-termini with GFP and/or mildly overexpressing N-terminal GFP fusions from the weak *nmt81* promoter (indicated by GFP before their name) were grown to mid-log phase at 25°C and imaged by confocal microscopy. *S. pombe* DUBs localize (A) exclusively to the nucleus, (B) to the nuclear envelope, (C) both to the nucleus and cytoplasm, (D) both to the nucleus and specific cytoplasmic structures, (E) exclusively to specific cytoplasmic structures (arrows denote localization to septa), or (F) diffusely in the cytoplasm. Bar: 5 µm for all panels.

**Figure 3:**
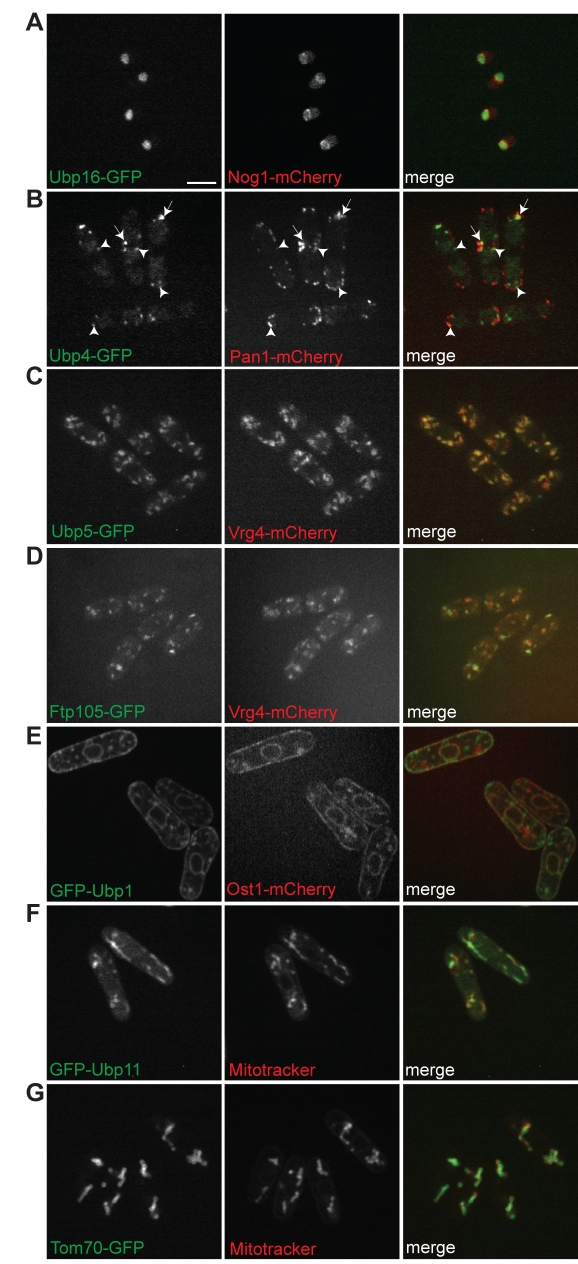
Localization of DUBs to different cellular compartments. (A–G) Strains expressing the indicated tagged proteins or stained with MitoTracker Red were grown to mid-log phase at 25°C and imaged by confocal microscopy. Images from the left and center panels are merged in the right panels. Co-localizing and adjacent endosomal structures are indicated with arrows and arrowheads, respectively. All proteins were endogenously tagged at their C-termini with GFP or mCherry except GFP-Ubp1 and GFP-Ubp11, which were expressed at low levels under the control of the *nmt81* promoter. Bar: 5 µm.

We examined the localization of all 20 putative *S. pombe* DUBs, endogenously tagged with green fluorescent protein (GFP) at their C-termini, as well as the localization of five of these DUBs tagged with GFP at their N-termini (Figure 2; Table 1). Ubp6, Ubp8, Ubp14, Ubp16, Rpn11, and Uch2 are exclusively nuclear (Figure 2A and 2B), while Uch1, Ubp12, Ubp15/Ubp21, Ubp9, and Otu1 are present both in the nucleus and the cytoplasm (Figure 2C and 2D). Ubp6, Ubp8, and Ubp14 are present in the nucleoplasm but excluded from the nucleolus (Figure 2A), whereas Ubp16 localizes exclusively in the nucleolus, where it co-localizes with the nucleolar marker Nog1 [30] (Figure 3A). As shown previously, Rpn11 and Uch2 localize primarily to the nuclear envelope (Figure 2B), where they interact with the proteasome [31],[32]. The existence of DUBs that localize to both the nucleus and the cytoplasm suggests that shuttling between the two compartments might regulate their activity. Moreover, the abundance of nuclear DUBs (both in terms of number and apparent concentrations as estimated by GFP intensity) highlights the importance of deubiquitination activity inside the nucleus, e.g., for proteasome function, COP9 signalosome function, histone deubiquitination and transcriptional regulation, cell cycle control, ubiquitin homeostasis, and DNA repair.

**Table 1:** Summary of the domain architecture, localization, and interaction profile of the *S. pombe* DUBs.

*S. pombe*		*S. cerevisiae*	*H. sapiens*				Complex Conservation		
DUB	UniProtKB	DUB	DUB	Domains^a^	Localization^b^	Interactors	*S. cerevisiae*	*H. sapiens*	References
Ubp1	Q9USM5	Ubp12p	NA	USP, DUSP	CS (ER)				
Ubp2	Q9P3U0	Ubp2p	NA	USP	C	Ucp6	+		48, 49
Ubp3	O94269	Ubp3p	USP10	USP	C	Nxt3	+	+	15, 51
Ubp4	O60139	Doa4p, Ubp5p	USP8	USP	CS (endosomes)	Sfp47			
Ubp5	Q09879	Ubp15p	USP7	USP, MATH	CS (Golgi)	Ftp105			
Ubp6	Q92353	Ubp6p	USP14	USP, UBL	N	26S proteasome	+	+	10, 43
Ubp7	Q9P7S5	Ubp11p	USP45	USP	C				
Ubp8	Q09738	Ubp8p	USP22	USP, ZnF-UBP	N	SAGA subunits	+	+	11, 12
Ubp9	Q9P7V9	Ubp9p, Ubp13p	USP12, USP46	USP	N, CS (cell tips, septum)	Bun62, Bun107	+	+	18, 76, 77, 78, 79
Ubp11	Q9UUD6	NA	NA	USP	M	Tom70			
Ubp12	O60079	Ubp12p	USP4, USP15	USP, DUSP	N, C				
Ubp14	Q11119	Ubp14p	USP5	USP, ZnF-UBP, UBA	N				
Ubp15	Q9UTT1	Ubp15p	USP7	USP, MATH	N, CS				
Ubp16	O74442	Ubp10p	NA	USP	No				
Uch1	Q10171	Yuh1p	UCHL3	UCH	N, C				
Uch2	Q9UUB6	NA	UCHL5	UCH	NE	26S proteasome		+	45, 46, 47
Otu1	O13974	Otu1p	YOD1	OTU	N, C	Cdc48	+	+	18, 54, 56
Otu2	Q9UUK3	Otu2p	OTUD6B	OTU	C				
Sst2	Q9P371	NA	STAMBP	JAMM	CS (endosomes)				
Rpn11	P41878	Rpn11p	PSMD14	JAMM	NE	26S proteasome	+	+	6, 7

**^a^**Domains are as defined in Figure 1 legend.

**^b^**C, cytoplasmic; CS, cytoplasmic structure; N, nuclear; NE, nuclear envelope; No, nucleolus; M, mitochondria.

NA, not applicable.

Seven *S. pombe* DUBs localize to distinct cytoplasmic structures or organelles (Figure 2D and 2E). In addition to localizing to the nucleus, Ubp9 localizes to septa and cell tips (Figure 2D). Ubp4, Ubp5/Ubp22, Sst2, and Ubp15 (also nuclear) localize to cytoplasmic spots reminiscent of vesicular structures. Ubp4-positive structures are adjacent to early endocytic sites, labeled with Pan1, suggesting that these structures are indeed endosomes (Figure 3B) [33]. This is consistent with the fact that USP8/UBPY, the mammalian homolog of Ubp4, interacts with endosomal sorting complex required for transport (ESCRT) components on multi-vesicular bodies [34]. Sst2/AMSH is another DUB that interacts with ESCRT components in mammalian cells [35],[36]. Multi-vesicular body sorting is defective in *sst2*-null *S. pombe* cells [22],[34],[36], suggesting that Sst2-positive structures (Figure 2E) are also endocytic. In addition, Ubp4, Sst2, and Ubp15, as well as Ubp9, localize to septa (Figure 2D and 2E), a site of active endocytosis in *S. pombe* [37], indicating an important role for deubiquitinating activity during cell division. Co-localization of Ubp5 with Vrg4, a Golgi protein [38] (Figure 3C), shows that Ubp5 is the first yeast DUB, to our knowledge, detected mainly at Golgi cisternae. Ubp1 (visualized best with an N-terminal tag) localizes to the ER (Figure 2E), as shown by its co-localization with Ost1 [39] (Figure 3E), whereas Ubp11 localizes to mitochondria (Figures 2E and 3F). Finally, Ubp2, Ubp3, Ubp7, and Otu2 exhibit a diffuse cytoplasmic localization (Figure 2F); it is possible that one or more of these DUBs is involved in scavenging ubiquitin that has been trapped in inactivated forms in the cytoplasm ([3] and references therein).

### Identification of DUB Complexes Using Proteomics

**Figure 4:**
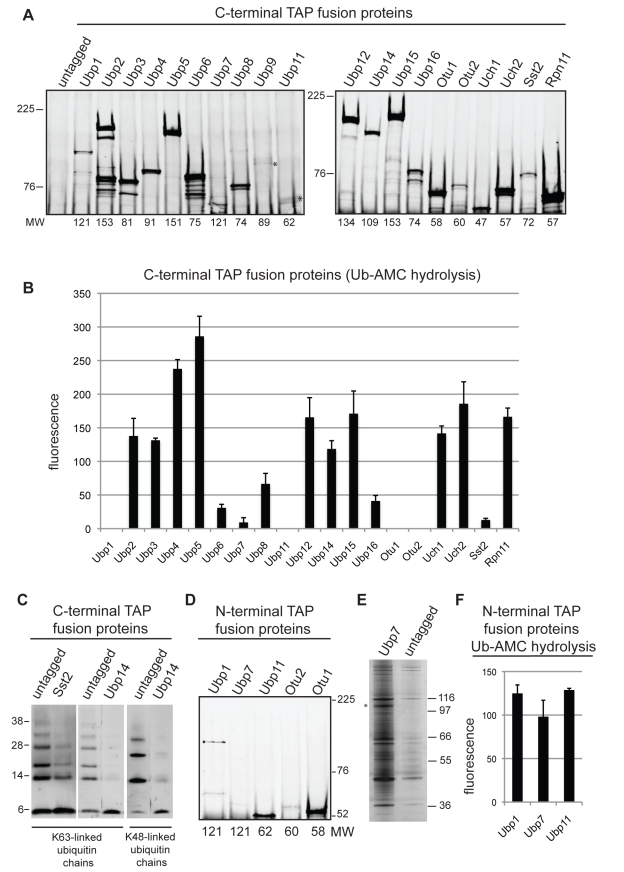
Enzymatic activity of *S. pombe* DUBs. (A) Equivalent amounts of cells expressing C-terminally TAP-tagged DUBs were lysed under native conditions, the TAP-tagged proteins were immunoprecipitated, and detected by immunoblotting. Asterisks indicate the fainter bands corresponding to Ubp9 and Ubp11. The expected molecular weight (MW, in kilodaltons) of the TAP-tagged DUB is provided below each lane. (B) DUB activities of the DUB-TAP immunoprecipitates were analyzed using Ub-AMC as a substrate. Data are mean ± SEM of two independent experiments. (C) DUB activities of Sst2-TAP and Ubp14-TAP immunoprecipitates were analyzed using K63- and/or K48-linked ubiquitin chains as substrates. (D and E) Equivalent amounts of cells expressing low levels of N-terminally TAP-tagged DUBs from the *nmt81* promoter were lysed under native conditions, and the TAP-tagged proteins were detected by (D) IP and immunoblotting or (E) silver staining. Ubp7 is indicated by an asterisk. (F) DUB activities of N-terminally tagged proteins were assayed using Ub-AMC as a substrate. Data are mean ± SEM of two independent experiments.

The *S. pombe* DUB localization data indicate that deubiquitination takes place in multiple cellular compartments. To address how DUBs might be targeted to, and regulated at, these discrete subcellular locations, we performed a comprehensive proteomic analysis of these enzymes using endogenously tandem affinity purification (TAP)–tagged forms of all 20 *S. pombe* DUBs. We purified the DUBs using TAP and detected interacting partners by 2D liquid chromatography–tandem mass spectrometry (LC-MS/MS). The DUB-TAP constructs we used were detectable by immunoprecipitation (IP) followed by immunoblotting (Figure 4A), except for Ubp7, which was detected by silver staining and LC-MS/MS after the TAP purification, but does not appear to transfer efficiently to polyvinylidine fluoride membranes under our experimental conditions (Figure 4E). Moreover, 14 of the TAP C-terminal fusion proteins displayed DUB activity towards the DUB artificial substrate ubiquitin 7-amido-4-methylcoumarin (Ub-AMC; Figure 4B) or polyubiquitin chains (Figure 4C), showing that DUB activity was not compromised in these cases. For the C-terminal DUB-TAP fusion proteins that did not have detectable in vitro enzymatic activity we constructed N-terminal fusion proteins expressed at low levels under the control of the weak *nmt81* promoter. Three of the N-terminal TAP fusion proteins (Ubp1, Ubp7, and Ubp11; Figure 4D) were able to hydrolyze Ub-AMC (Figure 4F). Thus, we purified them using the N-terminal TAP epitope and included them in our proteomic analysis.

Each TAP/LC-MS/MS analysis was performed in duplicate, and the results are summarized in Table S1. Only proteins detected in both biological replicates are included. In addition, nonspecific proteins (false-positive interactors) identified in background runs or in over 50% of other unrelated TAP/LC-MS/MS analyses performed in our laboratory are denoted by gray shading in Table S1.

#### DUB interactions in macromolecular complexes

We recovered nine stable molecular complexes including the transcriptional co-activator SAGA and the 26S proteasome (Figures S3 and 5; Tables 1 and S1). The validity of our approach and analysis is substantiated by the fact that we identified known interactions of Ubp8 with the SAGA complex, and Rpn11, Uch2, and Ubp6 with the 19S regulatory particle of the proteasome [23],[28],[32],[40]. Under our experimental conditions Ubp8 co-purified with four SAGA subunits—Sus1, Tra1, Sgf73, and Sgf29 (Figure S3; Table S1). In *Saccharomyces cerevisiae,* three of these SAGA components, namely Ubp8p, Sus1p, and Sgf73p, are part of the histone H2B deubiquitinating module [41],[42], suggesting that *S. pombe* Ubp8 is similarly anchored to the SAGA complex.

**Figure 5:**
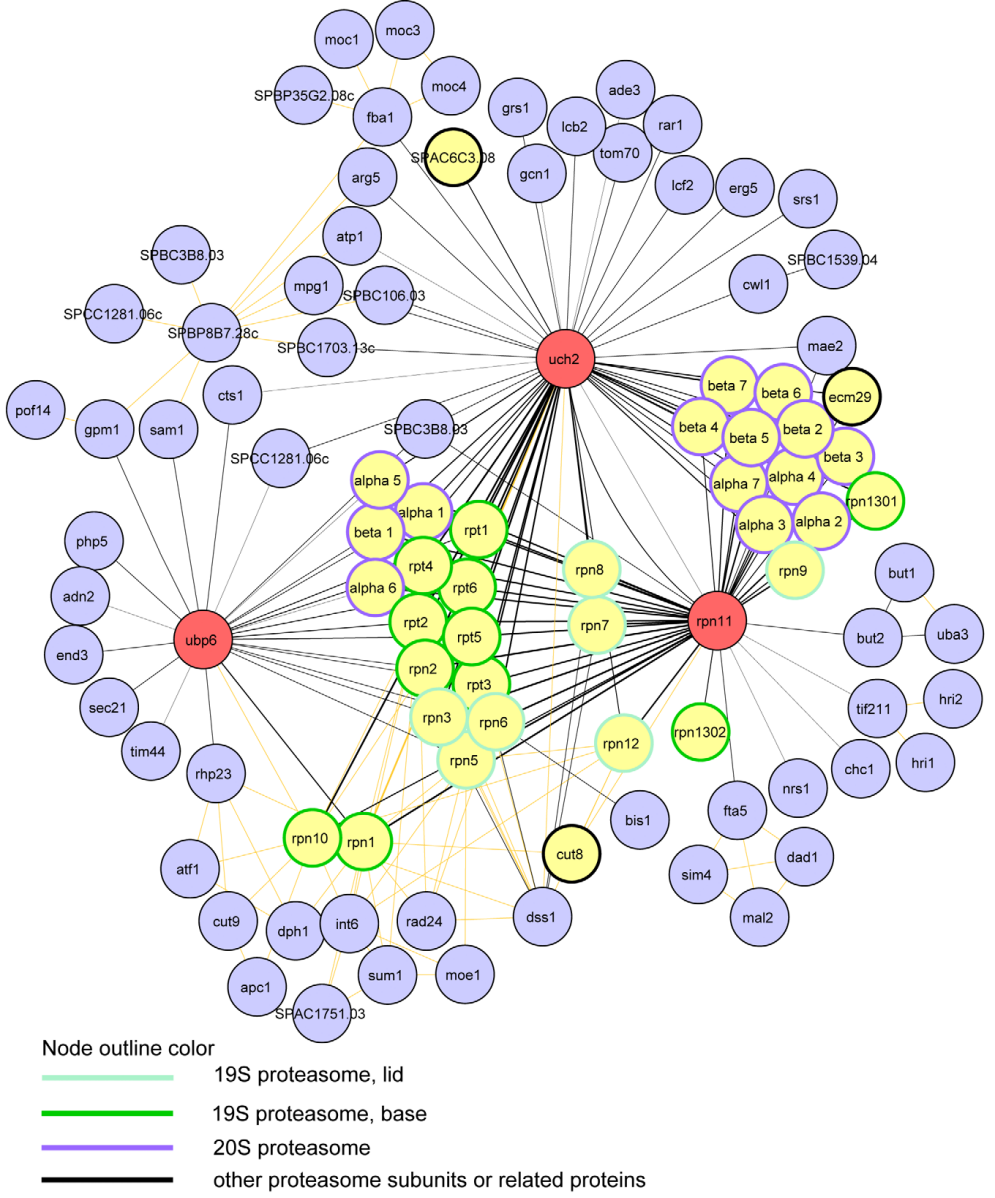
Network diagram of physical interactions of the proteasomal DUBs. Diagram of proteins identified by TAP/LC-MS/MS of Rpn11, Uch2, and Ubp6 and their interactions as curated in BioGRID (see Materials and Methods for details). DUB nodes are red, proteasome subunits and associated proteins are yellow (top MS hits in terms of TSC and validated by reciprocal Rpn11 and Uch2 TAPs), and all other protein nodes are blue. BioGRID interaction edge lines are shown in light orange, and TAP/LC-MS/MS edges are in black. TAP/LC-MS/MS edge line widths are coded according to TSC (thicker lines denote more spectral counts). The border color of the proteasomal nodes is as denoted in the key.

Proteasome components were the major interactors identified in three DUB purifications—Rpn11, Uch2, and Ubp6 (Figure 5). Rpn11 and Uch2 co-purified all of the 26S proteasome subunits (19S regulatory particle and 20S core particle) in quantities similar to those of the bait (Table S1), but the abundance of the proteasomal subunits identified in the Ubp6 TAP corresponded to approximately 1%–3% of the bait. The most abundant subunit to co-purify with Ubp6 was Rpn1/Mts4, its receptor at the base of the regulatory particle [23],[43]. As determined from studies in *S. cerevisiae*, Ubp6 is loaded on the proteasome under conditions of ubiquitin stress [44], which was not the case for our experiments and likely explains why Ubp6 co-purified so little of the regulatory particle. This result is in line with our Ubp6-GFP localization data showing that, under normal growth conditions, Ubp6 does not predominantly localize to the nuclear envelope (Figure 2A), where the proteasome is located (Figure 2B) [31],[32].

Comparison of the mass spectrometry (MS) results from purifications and interaction networks of Rpn11 and Uch2 (Figure 5; Table S1) revealed three previously unidentified *S. pombe* proteasome components (SPAC1782.01, SPBC342.04, and SPCC16A11.16c). The SPAC1782.01 ORF encodes a homolog of *S. cerevisiae* Ecm29p, a subunit that tethers the regulatory particle to the core particle [43]. In Uch2 and Rpn11 purifications, SPAC1782.01 (Ecm29) was found in amounts similar to that of other proteasomal components, suggesting that it is a functional homolog. SPBC342.04 and SPCC16A11.16c both have an ARM1 domain, also found in *S. cerevisiae* Rpn13p and *Homo sapiens* Rpn13 (ADRM1), suggesting that these proteins are the *S. pombe* Rpn13 homologs. SPBC342.04 (Rpn1301), SPCC16A11.16c (Rpn1302), and human Rpn13 share a C-terminal domain absent in *S. cerevisiae* Rpn13p (Figure S2). In human cells, this domain serves as the receptor for UCH37 (UCHL5) (*S. pombe* Uch2), a deubiquitinating enzyme absent from the *S. cerevisiae* genome [45]–[47]. Only Rpn1301 co-purifies with Uch2, suggesting that it is the receptor for Uch2 (Figure 5; Table S1).

#### Small DUB-containing complexes

**Figure 6:**
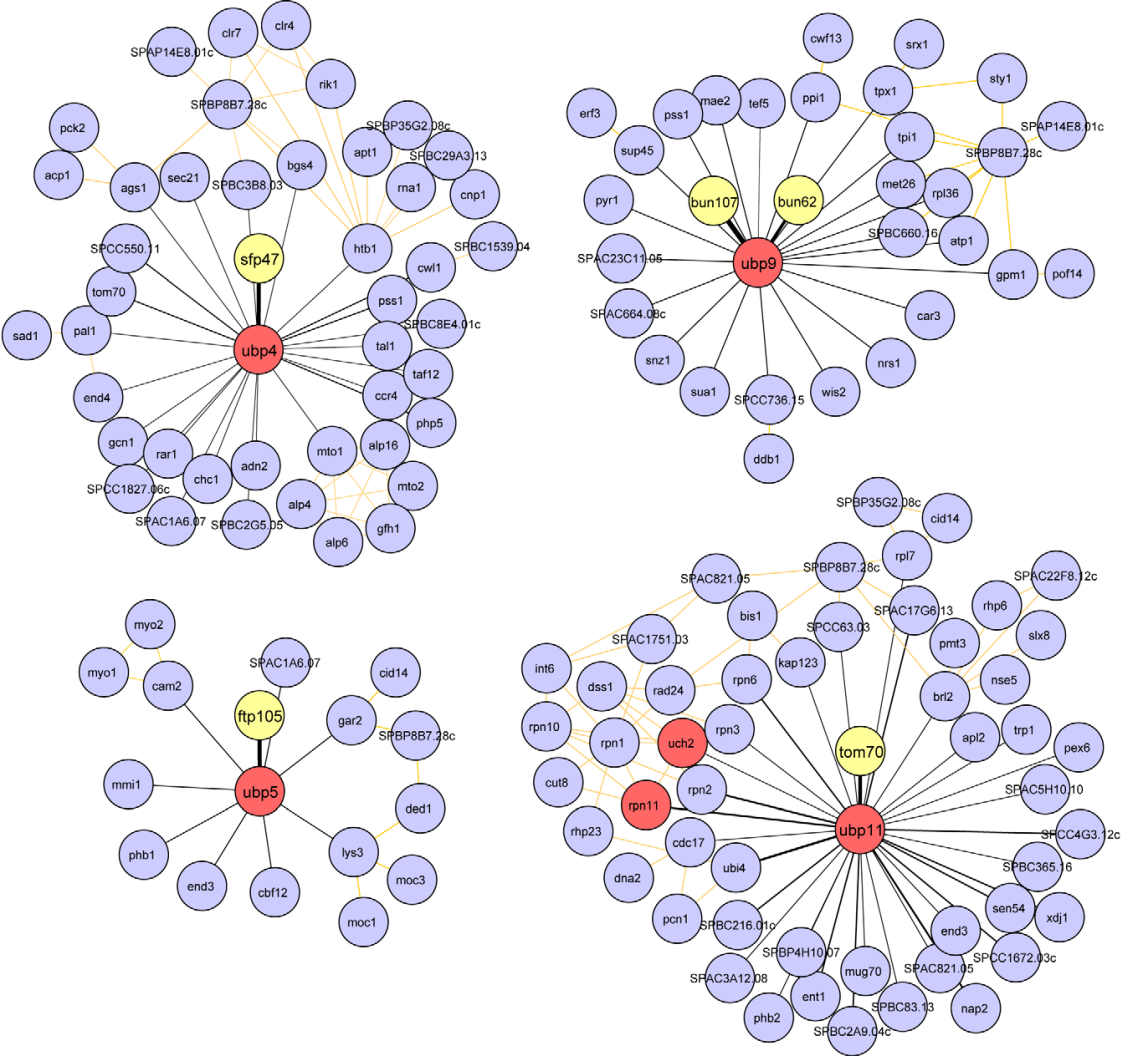
Network diagrams of physical interactions of new DUB protein complexes identified by TAP/LC-MS/MS. Diagrams were generated as described in Materials and Methods. DUB nodes are red, validated interactors (top MS hits in terms of TSC and confirmed by co-IP and/or reciprocal TAP) are yellow, and all other nodes are blue. Edges are colored and coded as in Figure 5.

In addition to the macromolecular complexes discussed above, our analysis revealed the presence of smaller DUB-containing protein complexes (Figures 6, S4, and S5; Table S1). In total, we identified seven smaller DUB-containing complexes. Three complexes previously described in *S. cerevisiae* and/or *H. sapiens* were also detected in *S. pombe*; these interactions include (1) Ubp2 and the UBA-domain-containing protein Ucp6 (Figure S4), (2) Otu1 and the Cdc48 AAA ATPase (Figure S4), and (3) Ubp3 and the ubiquitin protease cofactor Nxt3 (Figure S5). The Ubp2p–Ucp6p interaction is conserved in *S. cerevisiae* [48],[49]. Bre5p, the *S. cerevisiae* ortholog of Nxt3, is a co-factor essential for Ubp3p activation in vesicle transport and autophagy in *S. cerevisiae* [15],[16],[50], and this interaction is conserved in mammalian cells [18],[51]. Finally, Otu1 co-purifies with Cdc48, an AAA ATPase involved in delivering substrates to the 26S proteasome that is essential for the ER-associated degradation of misfolded proteins [52],[53]. *S. cerevisiae* Otu1p also physically interacts with Cdc48p [54] and human CDC48 (VCP/p97) binds to VCIP135, an OTU domain DUB [55]. Moreover, human CDC48 (VCP/p97) was recently reported as an interactor of YOD1, the human ortholog of Otu1 [18],[56]. Our data indicate that the Otu1–Cdc48 interaction is conserved among eukaryotes.

Four previously uncharacterized DUB complexes were identified by our proteomic approach (Figure 6; Table S1). First, Ubp4 co-purifies with a previously uncharacterized, nonessential protein encoded by the chromosomal locus SPAC7D4.02c. We named this 46.7-kDa SH3 domain protein Sfp47 for “SH3 domain Ubp4 partner of 47 kDa.” Second, Ubp5 co-purifies a 105-kDa nonessential protein encoded by the chromosomal locus SPAC17A5.16, which contains a Dymeclin domain (PFAM 09760) conserved from fungi to humans. SPAC17A5.16 contains five or six putative transmembrane helices that are conserved in its human homolog (see Discussion and Figure S18). Thus, we named this protein Ftp105 for “Ubp5 potential transmembrane protein of 105 kDa.” Third, Ubp9 co-purifies two nonessential WD repeat–containing proteins encoded by the chromosomal loci SPAC31A2.14 (8 WD repeats, 107 kDa) and SPAC12B10.03 (6 WD repeats, 62 kDa). We named these two proteins Bun107 and Bun62 for “binding Ubp9 of 107 and 62 kDa,” respectively. Lastly, we identified an interaction between Ubp11 and Tom70, a translocase of the outer mitochondrial membrane conserved in eukaryotes and not previously linked to the ubiquitin pathway (Figures 3G and 6; Table S1).

**Figure 7:**
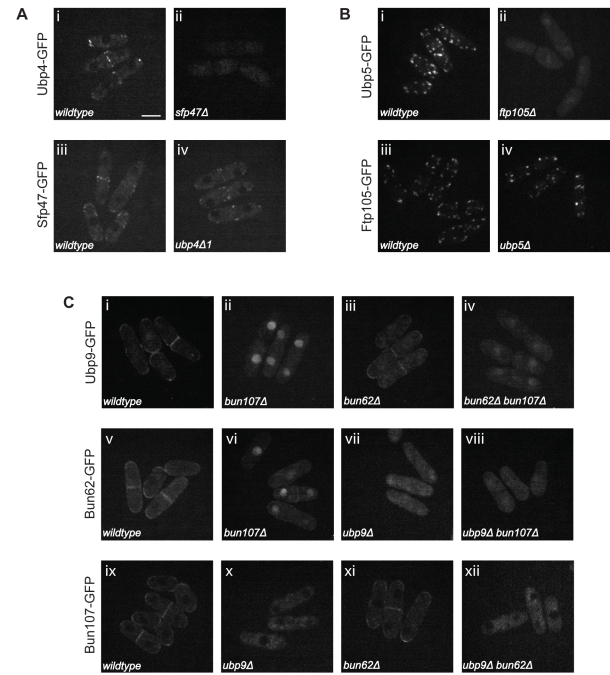
Localization of Ubp4, Ubp5, and Ubp9 depends on their partners. (A–C) Live cell imaging of the indicated endogenously tagged proteins in the indicated genetic backgrounds. Bar: 5 µm for all panels.

The aforementioned interactions are likely to be stoichiometric, since in all cases the putative interactor and the bait are detected in similar amounts, as reflected by the number of total spectral counts (TSC) (Table S1). We also identified putative interactors with lower spectral counts, which may indicate weaker or substoichiometric interactions with DUBs (Figures 5, 6, and S3–S11). Although some of these interactions may be specific, we did not consider them for further functional analysis because of their low relative abundance. However, we did investigate the role of the DUB family in *S. pombe* protein interactions using network diagrams (our TAP/LC-MS/MS results integrated with curated interactions in BioGRID; Figures 5, 6, and S3–S11). This analysis revealed a number of DUB interactions within protein networks that may provide insight into their cellular function. For instance, the putative endocytic DUB Ubp15 interacts with the binding partners of Ubp9 (Bun62 and Bun107), which localize at septa and cell tips (Table S1; Figures 7C and S9), and Pob1, an essential peripheral membrane protein involved in cell separation that partially co-localizes with Ubp15 on septa (Figure S12) [57]. A subpopulation of Ubp15 may be anchored by these interactors to these important sites of endocytosis during cell division, and its activity may be modulated by binding specific partners. One other intriguing observation is that Ubp1, an ER-associated DUB, interacts with four proteins (Snf21, Ssr1, Sif2, and Sif3) that tie it to the SWI/SNF and RSC complexes (Figure S6). Snf21 and Ssr1 are components of the SWI/SNF and/or RSC complexes themselves [58],[59], but Sif2 and Sif3 are linked to the complex via their interactor, Sad1 (thus they are named Sad1-interacting factors), suggesting that Ubp1 may function in chromatin remodeling [60]. Although we do not know the mechanism of nuclear import, overexpression of Ubp1 results in nuclear localization, supporting the idea that it may have a role inside the nucleus (data not shown).

### Validation of Novel DUB Complexes

To confirm the new binding interactions for Ubp4, Ubp5, Ubp9, and Ubp11, we performed co-IP and reciprocal TAP experiments using the potential DUB interactors as baits. Ubp4, Ubp5, and Ubp11 co-immunoprecipitate their partners Sfp47, Ftp105, and Tom70, respectively (Figure S13A and S13B). Ftp105-TAP also co-purified Ubp5 (Table S2), but the Sfp47-TAP construct was unstable and not useful for confirming an interaction with Ubp4. Each WD-repeat-containing (Bun) protein co-purified with Ubp9 and the other Bun protein in similar amounts (Tables S1 and S2), suggesting that the Ubp9–Bun62–Bun107 complex is stoichiometric. As expected, Ubp9, Bun62, and Bun107 co-immunoprecipitate the other two components of the complex in a wild-type background (Figure S13D, lanes 1–4 and 6–9).

### Regulation of DUB Activity and Localization by Interactors

Recent studies indicate that deubiquitinating enzymes can be regulated through their association with non-catalytic protein subunits. For example, *S. cerevisiae* Ubp6p activity is enhanced upon binding to Rpn1p (*S. pombe* Rpn1/Mts4), a proteasomal base subunit [43]. Similarly, UCH37, the human homolog of *S. pombe* Uch2, is activated by Rpn13, another proteasomal base subunit [45]–[47]. Therefore, we assessed how Ubp4, Ubp5, and Ubp9 activities are modulated upon formation of their respective complexes.

We first examined whether the binding partners influence DUB localization. For this purpose we deleted the genes coding for Ubp4, Ubp5, Ubp9, or their interactors. The levels of each DUB or interactor in wild-type and null mutants were quantitated on an Odyssey instrument and found not to change by more than 25% in any case (Figure S14A–S14C). Ubp4 and Sfp47 display a punctate localization on vesicular structures (Figure 7A). However, in an *sfp47-*null mutant Ubp4 localization is diffuse (Figure 7A). In contrast, Sfp47 localization is not affected by *ubp4Δ1* deletion (Figure 7A), indicating that Sfp47 recruits Ubp4 to endosomes, but not vice versa. Ubp5 and Ftp105 co-localize on vesicular structures overlapping with Golgi cisternae (Figures 3C, 3D, and 7B). In *ftp105*-null mutants, Ubp5 localizes diffusely in the nucleus and cytoplasm, but this is not the case for Ftp105, which localizes independently of Ubp5 (Figure 7B). Thus, similar to Sfp47, Ftp105 recruits Ubp5 to a specific cell compartment. Ubp9 and Bun62 localize to the nucleus, septa, and cell tips, while Bun107 localizes to septa and cell tips but is excluded from nuclei at steady state (Figure 7C). Ubp9 and Bun62 localization depends on Bun107, because in *bun107*-null mutant cells Ubp9 and Bun62 are predominantly nuclear (Figure 7C). Conversely, Ubp9 controls the localization of the other two components, which localize diffusely in the cytoplasm in *ubp9*-null cells (Figure 7C), even though their abundance is not significantly altered (Figure S14C). Thus, the localization of the Ubp9–Bun62 module and Bun107 is interdependent.

These localization data are consistent with biochemical analysis of the Ubp9 complex. Bun107 is not required for the Ubp9–Bun62 interaction as this sub-complex is still detected in a *bun107-*null mutant background. However, in *bun62-* or *ubp9*-null mutants the Ubp9–Bun107 and Bun62–Bun107 interactions, respectively, are disrupted (Figure S13D, lanes 5 and 10). These findings indicate that Ubp9 and Bun62 most likely form a pre-complex essential for the association with Bun107 and for cytoplasmic retention. Of note, the localization of Ubp9, Bun62, and Bun107 is regulated by Crm1-mediated nuclear export, since all three components are predominantly nuclear after leptomycin B treatment (data not shown). This suggests that even Bun107, the cytoplasmic anchor of the ternary complex, is shuttling between nucleus and cytoplasm, although its dynamic equilibrium is largely shifted towards the cytoplasm under physiological conditions. Moreover, we found that in wild-type cells, Ubp9 is phosphorylated, accounting for its variable SDS-PAGE mobility (Figure S14D), but this modification is lost in *bun62-* or *bun107*-null mutants (Figure S14C, lanes 1–4).

**Figure 8:**
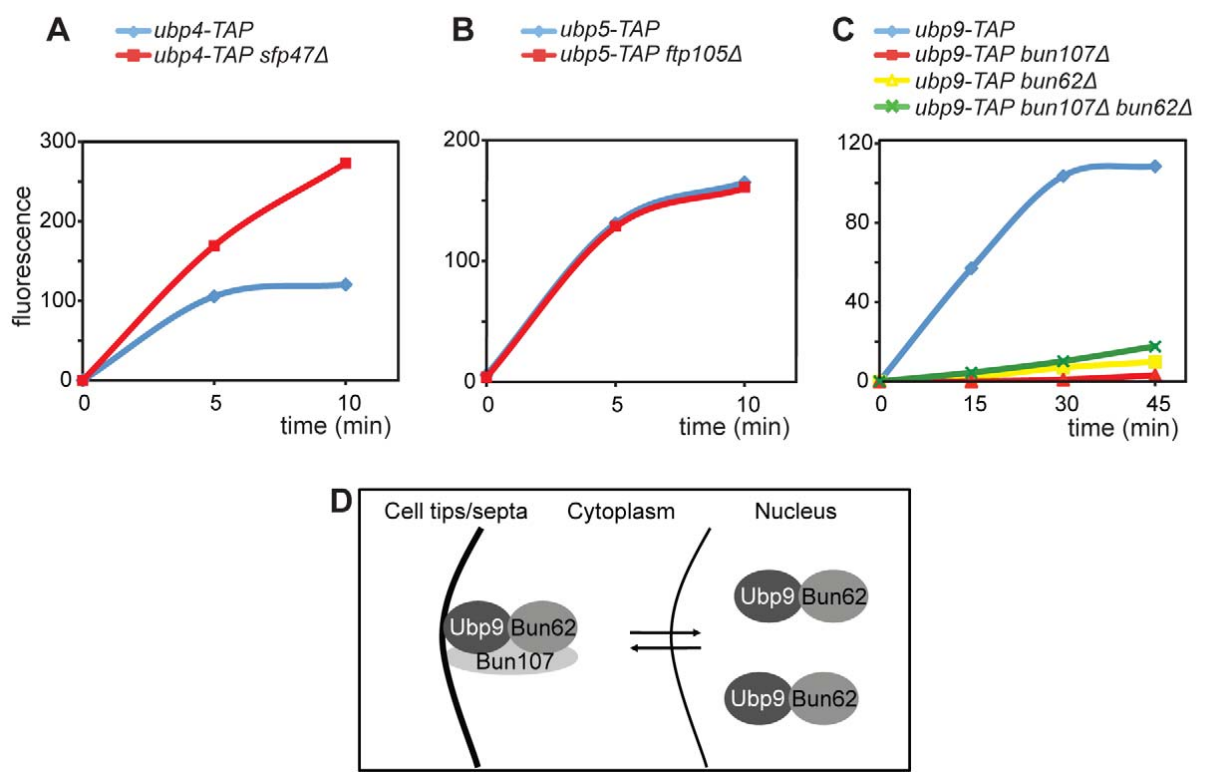
The activities of Ubp4, Ubp5, and Ubp9 are regulated by their partners. (A) Ubp4-TAP, (B) Ubp5-TAP, and (C) Ubp9-TAP were immunoprecipitated from either wild-type or the indicated *sfp47, ftp105, bun107,* and/or *bun62* deletion strains. Immunoprecipitates were assayed for deubiquitinating activity using Ub-AMC as a substrate. Alanine substitutions of the catalytic cysteine residues were also created in the *ubp4, ubp5,* and *ubp9* genes, and each was tagged at its endogenous locus with TAP so that these were the only versions of these DUBs produced by the genome. AMC fluorescence from control immunoprecipitates (enzymatically inactive DUBs) was subtracted, and the data were normalized according to protein quantities. (D) Working model for Ubp9 regulation by Bun107 and Bun62.

Next, we determined if the enzymatic activities of Ubp4, Ubp5, and Ubp9 are regulated by their interacting partners using the artificial substrate Ub-AMC. All of the above enzymes display DUB activity towards Ub-AMC (Figures 4 and 8), but their activity is affected differently by their interactors. Namely, Ubp5 activity is not significantly altered in *ftp105*-null cells, signifying that Ftp105 functions in recruitment of Ubp5 to the Golgi but not in its activation (Figure 8B). On the other hand, Ubp4 activity is enhanced in the absence of Sfp47 (Figure 8A), suggesting that Sfp47 recruits Ubp4 to endosomes where either Sfp47 itself or some other factor functions as an inhibitor. In contrast, Ubp9 is active only when in complex with both interactors (Figure 8C), demonstrating that the Ubp9–Bun62–Bun107 complex is required not only for Ubp9 recruitment to septa and cell tips but also for its enzymatic activity at these specific locations. A model of the dynamic localization of the Ubp9 DUB complex is presented in Figure 8D.

### Redundant DUB Activities Impact Endocytosis

The presence of Ubp9–Bun62–Bun107 at septa and cell tips suggests that this complex might be involved in endocytosis. To test this hypothesis we examined genetic interactions between *ubp9* and *end4/sla2, myo1,* and *wsp1,* which all have roles in cortical actin organization and endocytosis, two processes known to be interrelated in yeast cells [61]–[63]. Indeed, we observed that *ubp9Δ end4/sla2Δ* double-deletion mutant grows slower than the *end4/sla2Δ* simple mutant (Figure S15A). Moreover, *ubp9Δ wsp1Δ* and *ubp9Δ myo1Δ* double mutations are lethal at 36° C. Wsp1 and Myo1 activate the Arp2/3 complex, a known actin nucleator [63]. When actin polymerization is inhibited by Latrunculin B, the growth of *ubp9Δ wsp1Δ* and *ubp9Δ myo1Δ* double mutants is severely affected compared to single mutants (Figure S15B). FM4-64 internalization is decreased in *ubp9Δ myo1Δ* double-mutant cells as compared to the single mutants (Figure S15C and data not shown). Interestingly, *ubp9Δ myo1Δ* cells have prominent polarity defects, as shown by their aberrant cell shapes (Figure S15C). Together, these data show that Ubp9 is involved in regulating actin dynamics and/or endocytosis at cell tips and septa.

Although *ubp9Δ myo1Δ, ubp9Δ wsp1Δ*, *ubp9Δ sla2Δ* double-mutant cells display clear endocytosis and/or polarity defects (Figure S15), the *ubp9Δ* single mutant (Figure S15A) and the *ubp9Δ bun62Δ bun107Δ* triple mutant (data not shown) do not have growth defects. This is very likely due to a high degree of redundancy among the DUBs [64], and suggests that elucidating the role of any DUB might only be possible in a genetic context where many redundant DUB activities are “silenced.” Therefore, we set out to delete multiple DUBs that localize to vesicular structures and might be expected to have overlapping functions.

**Figure 9:**
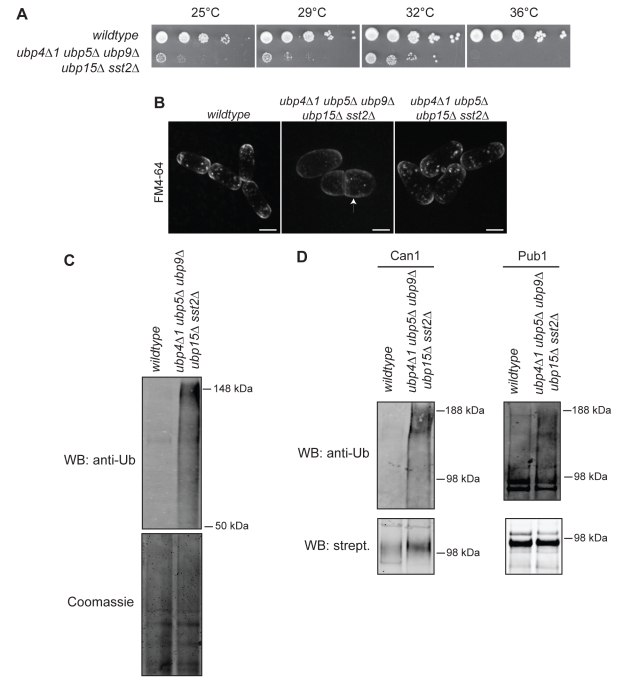
Redundant functions of Ubp4, Ubp5, Ubp9, Ubp15, and Sst2 in cell polarity and endocytosis. (A) Ten-fold dilution series of cells grown to mid-log phase were spotted on YE agar and grown at the indicated temperatures for 3 d. (B) Cells of the indicated genotypes grown to early log phase at 32°C and then shifted to 36°C for 3 h were labeled with FM4-64 for 6 min and imaged by confocal microscopy. The arrow indicates polarity defects of *ubp4Δ1 ubp5Δ ubp9Δ ubp15Δ sst2Δ* cells. Bar: 5 µm. (C) Anti-ubiquitin immunoblot and Coomassie staining of wild-type or *ubp4Δ1 ubp5Δ ubp9Δ ubp15Δ sst2Δ* cell lysates produced under fully denaturing conditions. WB: Western Blot. (D) Anti-ubiquitin and streptavidin immunoblots of Can1-HBH and Pub1-HBH purified from wild-type or *ubp4Δ1 ubp5Δ ubp9Δ ubp15Δ sst2Δ* cells under fully denaturing conditions.

The largest multiple mutant tested was the quintuple deletion *ubp4Δ1 ubp5Δ ubp9Δ ubp15Δ sst2Δ.* This strain displayed severe growth defects both at high and low temperatures (Figure 9A). It also displayed endocytosis and polarity defects, as shown by the decreased rate and number of ectopic sites of FM4-64 internalization, the small size of endosomal structures, and the aberrant cell shape (Figure 9B). Interestingly, the loss of all five DUB activities contributes to the severe growth phenotype, as none of the quadruple or triple mutant combinations was as defective as the quintuple mutant (Figure S16A and S16B). For example, the *ubp4Δ1 ubp5Δ ubp15Δ sst2Δ* strain does not display growth or endocytosis deficiencies (Figures 9B and S16A), suggesting that deletion of *ubp9* contributes significantly to the severe phenotype of the quintuple mutant.

To determine whether the growth and polarity defects correlated with increased ubiquitination of target proteins, cell lysates were produced under fully denaturing conditions and blotted for ubiquitin. There were increases in ubiquitinated proteins in triple- and quadruple-deletion mutants (Figure S16C), but the level of ubiquitinated proteins in *ubp4Δ1 ubp5Δ ubp9Δ ubp15Δ sst2Δ* cells was 20-fold increased compared to that in control cells (Figure 9C).

It has been well established that endocytic pathways are regulated by ubiquitination. Some targets of this modification include transmembrane nutrient receptors and regulators of the endocytic machinery [65]. Therefore, we tested whether the arginine transporter Can1 and the E3 ligase Pub1 (the ortholog of *S. cerevisae* Rsp5p) are similarly regulated by ubiquitination and whether their ubiquitination status is different in the *ubp4Δ1 ubp5Δ ubp9Δ ubp15Δ sst2Δ* mutant. Our in vivo ubiquitination assays using Histidine_6_-Biotin-Histidine_6_ (HBH) C-terminally tagged proteins show that polyubiquitinated Can1 levels are significantly increased in the quintuple mutant (Figure 9D). Pub1 is monoubiquitinated to the same extent in wild-type and *ubp4Δ1 ubp5Δ ubp9Δ ubp15Δ sst2Δ* cells, however, its polyubiquitination is 2.5-fold increased in the quintuple mutant (Figure 9D). Together, these results indicate that *S. pombe* Ubp4, Ubp5, Ubp9, Ubp15, and Sst2 DUBs contribute to the deubiquitination of both cargo and regulatory molecules during endocytosis.

## Discussion

In this work we provide a genome-wide analysis of *S. pombe* deubiquitinating enzymes. Our approach combining localization, proteomic, and enzymatic analysis of every *S. pombe* DUB has uncovered multiple modes of DUB regulation by non-catalytic partners via recruitment to a specific cellular compartment and/or modulation of activity. These studies show that a complex web of interactions govern the localization and activity of the DUB family.

In our study, the C-terminally GFP-tagged DUBs were expressed under their native promoters. For the enzymes that were not active as C-terminal fusions (Ubp1, Ubp7, Ubp11, Otu1, and Otu2) we also assayed their localization as N-terminal GFP fusion proteins and confirmed that the localizations were the same as the C-terminally tagged proteins (Figure 2). Matsuyama et al. previously provided localization data for the majority of *S. pombe* ORFs, including 18/20 of the DUBs presented here, but their DUB dataset is not identical to ours, most likely due to overexpression of the GFP-tagged proteins in their study [66]. GFP localization was most severely altered in the case of DUBs that we and others have shown to be part of protein complexes, such as Ubp5, Rpn11, Uch2, and Ubp9. For instance, Ubp5 localization to the Golgi apparatus depends on the presence of Ftp105. We suspect that when Ubp5 levels exceed those of Ftp105 because of *ubp5^+^* overexpression, Ubp5 is targeted to the nucleus [66], as is the case for endogenously GFP-tagged Ubp5 in *ftp105*-null mutant cells (Figure 7B).

Similar to the GFP localization studies, our proteomic analysis was performed using endogenously C-terminally tagged proteins in order to avoid perturbation of DUB protein–protein interactions. The validity of our results is bolstered by the fact that only proteins identified in both biological replicates were considered in the final analysis and by the stringent criteria we used for filtering (e.g., false discovery rate [FDR] <1%). Our analysis revealed that 55% of DUBs are involved in stable protein–protein interactions detectable both in asynchronous and mitotic cell cultures (Tables 1 and S1 and data not shown).

The activity assays we performed suggest that C-terminal fusion of *S. pombe* DUBs with a TAP tag does not interfere with their enzymatic activity in the majority of cases (14/20 DUBs; Figures 4 and 8). In some cases, little or no DUB activity was detected using the Ub-AMC substrate and/or polyubiquitin chains. Three of these enzymes were active as N-terminal TAP fusions (Ubp1, Ubp7, and Ubp11); thus, we also used these proteins for our proteomic analysis. Surprisingly, both OTU DUBs were inactive as both C-terminal and N-terminal fusion proteins, even though Otu1 co-purified with Cdc48, an interaction conserved both in *S. cerevisiae* and mammalian cells. We suspect that the loss of enzymatic activity is due to conformational changes that occur upon substrate binding that influence their affinity for ubiquitin [67], or that some factor present in the immunoprecipitate inhibits their activity. However, the low deubiquitination activity could also be due to our experimental conditions (e.g., protease inhibitors are used during cell lysis or low concentration of the DUB). Finally, Ubp6 C-terminal fusion protein was also inactive since, under our growth conditions, Upb6 is not robustly recruited to the proteasome, where it is activated by its receptor Rpn1/Mts4 [23],[43],[44].

### A Global Analysis of *S. pombe* DUB Localization

Our GFP localization data show that deubiquitination takes place in almost every cell compartment. More than 50% of the *S. pombe* DUBs, including the most abundant ones, localize to different compartments of the nucleus, whereas 35% localize to specific cytoplasmic structures (Figures 2A–2E and 3; Table 1). Almost 50% of the nuclear DUBs reside in the cytoplasm as well, suggesting that their transport to and from the nucleus might be regulated (Figure 2C and 2D). Nucleo-cytoplasmic shuttling of the mammalian DUB USP4 has been described [68], and it has also been shown that a subdomain within the USP domain of the DUB CYLD is necessary for its localization in the cytoplasm [69]. However, a role for interacting partners in regulating the nucleo-cytoplasmic transport of these proteins has not been reported. We have demonstrated that anchoring of Ubp5 to the Golgi and Ubp9 to cell tips/septa is mediated by their partners, Ftp105 and Bun107, respectively (Figure 7). These results define a mechanism for DUB cytoplasmic retention by interacting partners.

We also examined the localization of DUBs during the cell cycle using cells arrested in prometaphase via the *nda3-KM311* (β-tubulin) mutation or in S-phase after addition of hydroxyurea, a chemical agent that indirectly induces DNA damage. We did not observe any significant change in DUB localization (data not shown), suggesting that their recruitment to various cellular compartments, and especially their nucleo-cytoplasmic transport, is not strongly affected under these conditions.

### Examples of DUB Regulation

Exploration of several protein–protein interactions reported in this study have revealed new examples of DUB regulation. Ubp4 interacts with Sfp47, an SH3-domain-containing protein that is required for its localization to vesicular structures (Figures 6 and 7A). This finding is of particular interest, because Ubp4 homologs in *S. cerevisiae* and *H. sapiens*, Doa4p and USP8/UBPY, respectively, use very different endosomal “recruitment strategies.” The Doa4p N-terminus contains four conserved motifs that are required for its localization to endosomes, and its recruitment is mediated by its co-factor Bro1p, a component of the multi-vesicular body sorting machinery, which may also activate Doa4p [70],[71]. In contrast, Ubp4 enzymatic activity is reduced when targeted to endosomes by Sfp47, suggesting that some Ubp4 inhibitor analogous to Rfu1p, the Doa4p inhibitor in *S. cerevisiae,* might be present on this compartment (Figure 8A) [72]. On the other hand, human USP8 recruitment to the endosomes is dependent on its N-terminal MIT (microtubule interacting and transport) domain that associates with components of the ESCRT [73]. *S. pombe* Ubp4 does not have an extended non-catalytic N-terminus like Doa4p and USP8; however, it possesses a PXXP motif, which could mediate association with the Sfp47 SH3 domain (Figure S17). These results suggest that at least three independent mechanisms of DUB recruitment to endosomes have emerged during eukaryotic evolution, highlighting the importance of regulated deubiquitination in this compartment.

Another example of DUB regulation by localization is the recruitment of Ubp5 to the Golgi by Ftp105. Ftp105 contains five or six putative transmembrane helices (http://www.ch.embnet.org/software/TMPRED_form.html). To our knowledge, this is the first observation of a DUB being recruited to a compartment via interaction with a potential integral membrane protein. Ftp105 has a clear human homolog, C17orf28, “down-regulated in multiple cancers,” which is a putative tumor suppressor [26] (Figure S18). *ftp105* deletion results in Ubp5 mislocalization to the cytoplasm and the nucleus without affecting its activity. It would be interesting to explore whether other proteins containing domains of the dymeclin superfamily (PFAM 09742) have similar roles in other organisms, especially if they sequester DUBs by recruitment to specific structures, preventing them from functioning elsewhere. It is intriguing to note that Ubp5′s human ortholog, USP7/HAUSP, is a DUB regulating p53 and MDM2 stability and PTEN localization, as these proteins are associated with tumorigenesis and cancer progression [27],[74],[75].

Similar to Ubp5, the shuttling of Ubp9 between the nucleus and the cytoplasm and its anchoring at cell tips and septa are regulated by interaction with its WD repeat partners (Figure 7C). In contrast to Ubp5, the enzymatic activity of Ubp9 depends on its interaction with *both* partners (Figure 8C), indicating that Ubp9 is not functional in the nucleus and may be sequestered there. Ubp9 has clear orthologs in budding yeast (Ubp9 and Ubp13) and humans (USP12 and USP46) that interact with WD repeat proteins [18],[76]–[79]. Moreover, the human ortholog of Bun107 activates USP12 and USP46 [78]. Interaction of DUBs with WD repeat proteins is an intriguing new concept, as suggested by their abundance in human cells [18]. Ubp9 is intimately linked to the interrelated processes of cortical actin organization, endocytosis, and cell polarity in *S. pombe,* and it will be exciting to determine whether this mode of regulation and function is conserved in the *S. cerevisiae* and *H. sapiens* Ubp9 complexes.

### Redundant DUBs Involved in Endocytosis

Although multiple negative genetic interactions suggest that Ubp9 is involved in actin dynamics, endocytosis, and cell polarity, the *ubp9*Δ single mutant and the *ubp9*Δ *bun107*Δ *bun62*Δ triple mutant do not show any phenotypic abnormalities. This result is not surprising in the light of work done in *S. cerevisiae* that has shown that deletion of single or multiple DUBs results in only a mild or no growth phenotype [64]. Given the substantial functional overlap among these enzymes, it is obvious that exploring DUB function in yeast requires combination of multiple mutations. For that purpose we generated multiple mutants of DUBs residing on vesicular structures and revealed that five of these enzymes share a common function in maintaining cell polarity and endocytosis efficiency (Figure 9B). This approach allowed us to identify two endocytosis-related substrates of these enzymes and could be a powerful tool for the discovery of several other deubiquitination targets, especially ones involved in actin dynamics and cell polarity.

### Concluding Remarks

Recently, Sowa et al. reported a proteomic analysis of approximately 80% of putative human DUBs [18]. The putative human DUBs were overexpressed as N-terminal Flag-HA fusion proteins and purified by anti-HA IP, and proteins were detected using LC-MS/MS. Sixteen of the 20 *S. pombe* DUBs are conserved in *H. sapiens* and seven of the 16 appear to be involved in the same protein complexes in both organisms (Ubp3, Ubp8, Ubp9, Ubp6, Rpn11, Uch2, and Otu1) (Figures 5, 6, and S3–S5; Tables 1 and S1; [18]). Moreover, TAP-LC-MS/MS analysis of proteins containing nonfunctional or non-ubiquitin-specific DUB domains (Ubp10, Rpn8, eIF3f, and Csn5) shows that the human interaction networks are conserved in *S. pombe* (data not shown; [18]). However, the two datasets contain some important differences, namely: (1) USP4 and USP15, the human orthologs of Ubp12, seem to be part of a pre-mRNA processing module in human cells, but no such interaction is detected in *S. pombe;* (2) USP7, the human ortholog of Ubp5 and Ubp15, interacts with DNA damage modules in human cells, whereas *S. pombe* Ubp5 and Ubp15 are involved in membrane trafficking/polarity control, and Ubp5 is targeted to the Golgi by its partner Ftp105; (3) *S. pombe* Ubp4 interacts with an SH3 domain protein (Figures 6 and S13A), as does its human ortholog, USP8 [34], but Sowa et al. did not detect this interaction. Additionally, there were several E3 ligases detected in the human dataset, whereas only three were identified in our study (Ubp11 purification; Table S1). This might reflect some evolutionary divergence between human and *S. pombe* DUBs and/or may result from the many technical and analytical differences between the two studies. Finally, neither study was able to detect DUB substrates, likely because of the transient, dynamic nature of enzyme–substrate interactions.

Our genome-wide screen of *S. pombe* deubiquitinating enzymes allowed the detailed description of their subcellular localization, the identification of previously uncharacterized *S. pombe* protein complexes essential for DUB function, and the establishment of these family members as bona fide deubiquitinating enzymes. This combination of experimental approaches provides new insight into how the activity of deubiquitinating enzymes is finely tuned by non-catalytic partners. Some of the protein–protein interactions described here are conserved between *S. pombe*, *S. cerevisae,* and mammalian cells. This suggests that the modes of regulation and function assigned to these enzymes are likely valid in other organisms and highlights the usefulness of combined approaches and simple systems to understand complex biological phenomena.

## Materials and Methods

### Yeast Strains, Media, Genetic Methods, and Vector Construction

Yeast strains (Table S3) were grown in yeast extract (YE) medium. For expression of N-terminally tagged proteins, strains were transformed with pREP expression vectors, containing a thiamine-repressible promoter, using a standard sorbitol transformation procedure [80]. Transformed strains were first grown on minimal medium containing thiamine to suppress expression. To induce expression, cells were grown in liquid minimal medium lacking thiamine for 18h [81]. Cell cultures used for TAP purifications were grown in 2 l of 4× YE medium (C-terminally TAP-tagged proteins) or in 8 l of EMM supplemented with the appropriate nutrients (N-terminally TAP-tagged proteins). For in vivo ubiquitination assays, strains were grown in 100 ml of 4× YE medium. All 20 DUBs and *bun62, bun107, ftp105, sfp47, pob1, pan1*, *tom70, can1,* and *pub1* were tagged endogenously at the 3′ end with GFP, TAP, FLAG_3_, V5, mCherry, HBH, linker-TAP, or linker-GFP, as previously described [82]. The linker sequence in the linker-TAP and linker-GFP tag cassettes translates to ILGAPSGGGATAGAGGAGGPAGLI [83]. DNA coding for Ubp1, Ubp7, Ubp11, Otu1, or Otu2 was amplified by PCR from genomic *S. pombe* DNA. The PCR products were digested with the appropriate restriction enzymes (SalI/BamHI for Ubp1, NdeI/XmaI for Ubp7, Ubp11, and Otu2, and XmaI for Otu1), sublconed into pREP81-TAP and pREP81-GFP vectors, and verified by sequencing.

Disruption of genes (*ubp4, sfp47, ubp5, ftp105, ubp9, bun107, bun62,* and *sst2*) was achieved by PCR-based one-step homologous recombination [82], targeting the entire open reading frames. In the case of *ubp4*Δ*1,* however, only the sequence corresponding to amino acids 156–593 was removed because of the presence of previously undetected 5′ exons. These genes were targeted for deletion using *ura4^+^* as the selectable marker, stable integrants were selected, and the deletions were confirmed by PCR. A lithium acetate method was used for yeast cell transformations [84]. For gene replacement at the endogenous locus, the entire ORF plus at least 500 bp of 5′ and 3′ flanking nucleotides was sub-cloned into the pIRT2 vector containing the *leu2+* marker, and the mutations were inserted by site-directed mutagenesis and sequenced. Haploid strains (ubp4::ura4, ubp5::ura4 or ubp9::ura4) were transformed with pIRT2-*ubp4(C236S),* pIRT2-*ubp5(C222S),* or pIRT2-*ubp9(C50S),* stable integrants were selected by resistance to 5-Fluoroorotic acid (5-FOA), and the integrations were confirmed by PCR. Strain construction and tetrad analysis were accomplished through standard methods.

### Protein Methods

Cell pellets were frozen in a dry ice/ethanol bath and lysed by bead disruption in NP-40 lysis buffer under either native (Figures 4, 8, and S13) or denaturing (Figures 8D and S14A–S14C) conditions as previously described [85], except with the addition of 0.1 mM diisopropyl fluorophosphate (Sigma-Aldrich). Proteins were immunoprecipitated by anti-GFP (Roche), anti-V5 (Invitrogen), and anti-FLAG (M2; Sigma-Aldrich) antibodies and Protein G Sepharose beads (GE Healthcare) or IgG Sepharose beads (GE Healthcare). Immunoblot analysis was performed as previously described [86], except that secondary antibodies were conjugated to Alexa Fluor 680 (Invitrogen) and visualized using an Odyssey Infrared Imaging System (LI-COR Biosciences).

For in vivo ubiquitination assays (Figure 9D) *can1* and *pub1* were tagged at their endogenous C-termini with an HBH affinity tag. Tagged proteins were purified using a modified version of the two-step tandem affinity purification under fully denatured conditions [87]. For each strain, cell pellets were lysed by bead disruption into Buffer 1 (8 M Urea, 300 mM NaCl, 50 mM NaPO_4_, 0.5% NP40, and 4 mM Imidazole [pH 8]) and incubated with Ni-NTA agarose beads (Qiagen) for 4 h at room temperature. After incubation, beads were washed 4× with Buffer 3 (8 M Urea, 30 0mM NaCl, 50 mM NaPO_4_, 0.5% NP40, and 20 mM Imidazole [pH 6.3]) and eluted in Buffer 4 (8 M Urea, 200 mM NaCl, 50 mM NaPO_4_, 0.5% NP40, 2% SDS, 100 mM Tris, and 10 mM EDTA [pH 4.3]). The pH of the eluate was adjusted to 8 before adding streptavidin ultra-link resin (Pierce) and incubating overnight at room temperature. After the second incubation, streptavidin beads were washed 4× with Buffer 6 (8 M Urea, 200 mM NaCl, 2% SDS, and 100 mM Tris [pH 8]) and 1× with Buffer 7 (8 M Urea, 200 mM NaCl, and 100 mM Tris [pH 8]). Purified proteins were detected by immunoblotting using a ubiquitin anti-serum (Sigma) and fluorescently labeled streptavidin (LI-COR Biosciences).

For comparison of ubiquitinated protein levels (Figures 9C and S16C), 40 OD cell pellets were lysed by bead disruption into Buffer 1 (8 M Urea, 300 mM NaCl, 50 mM NaPO_4_, 0.5% NP40, and 4 mM Imidazole [pH 8]), and lysates were analyzed by immunoblotting (polyclonal anti-ubiquitin antibody, Sigma) and Coomassie blue staining to normalize for protein quantities. Protein quantification (ubiquitinated species as measured by anti-ubiquitin immunoblot/total protein as measured by Coomassie staining) was performed using the Odyssey v3.0 software.

For phosphatase collapse (Figure S14D), immunoprecipitated Ubp9-TAP was incubated with lambda phosphatase (New England Biolabs) in 25 mM HEPES-NaOH (pH 7.4), 150 mM NaCl, and 1 mM MnCl_2_ for 30 min at 30°C.

For DUB activity assays, cell pellets were lysed under native conditions as described above with some differences: NaCl concentration was increased to 300 mM in the NP-40 lysis buffer and TAP-tagged proteins were immunoprecipitated by tosylactivated Dynabeads (Invitrogen) coated with rabbit IgG (MP Biomedicals). Immunoprecipitates were washed 3× in lysis buffer and 3× in DUB assay buffer (see next section for DUB assay buffer composition). For TAPs, cells were lysed under native conditions and proteins were purified as described previously [88].

### MS Sample Preparation and Methods

After purification, DUBs were TCA precipitated and resuspended in 8 M Urea, 50 mM Tris (pH 8), reduced with Tris (2-caroxyethyl phosphine), alkylated with iodoacetamide, and digested overnight at 37°C with Trypsin Gold (Promega) after diluting to 2 M urea with 50 mM Tris (pH 8). MS was performed as previously described [89] with the following modifications. Peptides were loaded onto columns with a pressure cell and were separated and analyzed by three-phase multidimensional protein identification technology on a linear trap quadrupole instrument (Thermo Electron). An autosampler (FAMOS) was used for 12 salt elution steps, each with 2 µl of ammonium acetate. Each injection was followed by elution of peptides with a 0%–40% acetonitrile gradient except the first and last injections, in which a 0%–90% acetonitrile gradient was used. Eluted ions were analyzed by one full precursor MS scan (400–2,000 mass-to-charge ratio) and four tandem MS scans of the most abundant ions detected in the precursor MS scan under dynamic exclusion.

### MS Data Analysis

Centroided peak lists for MS2 spectra were extracted from THERMO RAW files using Scansifter v.2.1.1 (software developed in-house by Vanderbilt University Medical Center) and converted to DTA files. Spectra with less than six peaks were excluded from our analysis. If 90% or less of spectral intensity of a tandem mass spectrum was detected at *m*/*z* values lower than the precursor ion, then the precursor ion was assumed to be +1. All other spectra were processed using precursor charge states of +2 *and* +3. Protein identification was performed with the SEQUEST algorithm [90] (v.27, rev.12) on a high-performance computing cluster (Advanced Computing Center for Research & Education at Vanderbilt University) using the GeneDB/Sanger Institute *S. pombe* protein database, created October 2009. Contaminant proteins (e.g., keratin and IgG; 73 total) were added, and all database sequences were reversed and concatenated to allow estimation of FDRs (total of 10,186 entries). SEQUEST parameters were as follows: strict tryptic cleavage, maximum of ten missed cleavage sites, maximum of four amino acid modifications per peptide, allowed modification of cysteine (+57.05 for carboxamidomethylation) and methionine (+16 for oxidation), the average mass of precursor ions was required to fall within a 1.25-*m/z* window, and fragment ions were required to fall within 0.5 *m*/*z* of their monoisotopic masses. SEQUEST out files were converted to pepXML files by SQter (spectral data SEQUEST search results) [91] for analysis in IDPicker 2.4.0 [92],[93] using the following filters: maximum FDR per result, 0.01; maximum ambiguous IDs per result, 2; minimum peptide length per result, 5; minimum distinct peptides per protein, 5; minimum additional peptides per protein group, 2; indistinct modifications, M 15.994 C 57.05. Parsimony rules were applied to generate a minimal list of proteins to explain all of the peptides that passed our entry criteria. No reversed proteins passed our criteria so that zero proteins were estimated to be falsely identified in this list, i.e., a 0% FDR.

Duplicate DUB and negative control purifications (no TAP tag for C-terminal or empty pREP81-TAP for N-terminal) were processed as described above. Cross-species contaminant proteins (e.g., keratin) have been removed from all protein ID lists. In addition, only proteins identified in both biological replicates are included in the protein ID table (Table S1). Gray-shaded rows denote proteins identified in the negative controls or in over 50% of other unrelated TAP/LC-MS/MS analyses performed in our laboratory. Blue-shaded rows indicate proteins identified in over 50% of all the DUB purifications and, in the case of the N-terminal TAPs, proteins identified in all six N-terminal TAP purifications. This method of background estimation is likely conservative because at least two proteins identified with low spectral counts as “background” are also identified as interactors with high relative abundance to bait (Nxt3, Ubp3′s partner, and Rpt6, a proteasomal component); when Nxt3 and Rpt6 are present at high relative abundance to bait, they are shaded orange in Table S1 to denote this distinction. Yellow-highlighted rows indicate proteins that interacted with the bait (denoted by bold) that have been validated by co-IP and/or reciprocal TAP or reported in the literature for *S. pombe* DUBs or their homologs.

### Network Interaction Diagrams

We analyzed the networks of proteins identified in each of the duplicate DUB TAP/LC-MS/MS analyses (excluding background, unshaded rows in Table S1) using the *Schizosaccharomyces_pombe* BioGRID database v3.0.65 [94] and generating network diagrams using Cytoscape v2.7.0 [95]. Interactions between each protein identified in the DUB TAP/LC-MS/MS analyses were queried using the BioGRID Plugin 2.0 for physical interactions (4,007 total interactions in BioGRID for *S. pombe*) in Cytoscape. We merged the BioGRID interactions with our TAP/LC-MS/MS data to generate Figures 5, 6, and S3–S11. The edge widths of protein interactions identified by TAP/LC-MS/MS in Figures 5 and 6 are coded according to the TSC. The top MS hits (TSC)/validated partners (our study) and interactors reported in the literature for DUB homologs are highlighted in yellow and placed close to the DUB to mark this distinction.

### DUB Activity Assays

In vitro enzymatic assays with 1 µM Ub-AMC (Boston Biochem) were performed using the DUB-TAP IPs (left on dynabeads) in 50 µl of reaction buffer (20 mM HEPES-KOH [pH 7.8], 20 mM NaCl, 0.1 mg/ml BSA [Sigma-Aldrich], 0.5 mM EDTA, and 10 mM DTT) at 32°C for 15 min (Figure 4B), 50 min (Figure 4F), or the indicated times (Figure 8). Fluorescence was monitored in a Molecular Devices FlexStation 3 fluorometer after dynabeads were removed. Fluorescence corresponding to a control reaction (reaction mixture containing immunoprecipitate from untagged cells) was subtracted (Figure 4). For the analysis of Ub-AMC hydrolysis kinetics, the control reaction used for background fluorescence subtraction contained immunoprecipitate from strains encoding catalytically inactive DUBs (Ubp4 C236S, Ubp5 C222S, or Ubp9 C50S; Figure 8). To compare the enzymatic activities of Ubp4, Ubp5, and Ubp9 in different genetic backgrounds, the immunoprecipitates were analyzed by immunoblotting, and the proteins were quantified using the Odyssey v3.0 software, and fluorescence measurements (enzyme activity) were corrected for protein amount. In vitro enzymatic assays with polyubiquitin (Ub_1–7_) chains (Boston Biochem) were performed using the DUB-TAP IPs (left on dynabeads) in 20 µl of reaction buffer at 32°C for 4 h. K63-linked polyubiquitin (Ub_1–7_) chains (Sst2 DUB assay) (50 ng) were added to 20 µl of reaction buffer (50 mM Tris-HCl [pH 7.4], 25 mM KCl, 5 mM MgCl_2_, and 10 mM DTT). K48-linked or K63-linked polyubiquitin (Ub_1–7_) chains (Ubp14 DUB assay) (25 ng) were added to 20 µl of reaction buffer (50 mM Tris-HCl [pH 7.8], 25 mM KCl, 5 mM MgCl_2_, and 10 mM DTT). Reaction mixtures containing immunoprecipitate from untagged cells were used as negative controls. The reactions were stopped by addition of SDS sample buffer. Ubiquitin chains and monomers were analyzed by immunoblotting after dynabead removal, as described above.

### Microscopy Methods

Cells were grown to mid-log phase and imaged live at 25°C using a spinning disk confocal microscope (Ultraview LCI; PerkinElmer) with a 100× NA 1.40 Plan-Apochromat oil-immersion objective and a 488-nm argon ion laser (GFP) or a 594-nm helium neon laser (mCherry, FM4-64, MitoTracker Red). Images were captured on a charge-coupled device camera (Orca-ER; Hamamatsu Photonics) and processed using Metamorph 7.1 software (MDS Analytical Technologies). Z-section slices were 0.5 µm. Visualization of endocytosis with FM4-64 was essentially as described [96]. Briefly, cells were grown in YE medium to an optical density (OD600) of 0.5, harvested by centrifugation, resuspended at OD_595_ 3–5 and placed on ice for 10 min. FM4-64 stock solution (1.63 mM in DMSO; Molecular Probes) was added to 400 µl of cold cells to a final concentration of 8.15 µM. A small sample of cells was immediately transferred to a microscope slide at room temperature and imaged by confocal microscopy as described above. Visualization of mitochondria was performed as in Jourdain et al. [97]. Briefly, MitoTracker Red CMXRos (Molecular Probes) was dissolved in DMSO at a concentration of 1 mM and diluted in minimal medium to 1 µM. Mid-log phase cells were incubated with MitoTracker Red (final concentration 100 nM) for 30 min. Cells were washed 3× in minimal medium, before being transferred to a microscope slide and imaged by confocal microscopy as described above.

### Sequence Alignments

Sequence alignments were performed using the Multalin software (http://bioinfo.genopole-toulouse.prd.fr/multalin/multalin.html) [98].

### Accession Numbers

The GeneDB (http://old.genedb.org/genedb/pombe/) accession numbers for the previously unnamed proteins discussed in this paper are Bun107, SPAC31A2.14; Bun62, SPAC12B10.03, Ecm29, SPAC1782.01; Ftp105, SPAC17A5.16; Rpn1301, SPBC342.04; Rpn1302, SPCC16A11.16c; and Sfp47, SPAC7D4.02c. All the DUB UniProt accession numbers are provided in Table 1, and all DUB and interactor GeneDB accession numbers are provided in Table S1.

## Supporting Information

Figure S1 **Alignment of** ***S. pombe*** **proteins excluded from our study.** (A) The USP domain sequences of Ubp10 and Ubp13 were aligned with the USP domain of Ubp4. The catalytic residues in the Cys and His boxes, respectively, are highlighted in green. Ubp10 lacks the catalytic cysteine but has an intact histidine box. Ubp13 lacks both catalytic boxes. (B) The JAMM domain sequences of Rpn8, Spp42, eIF3f, and eIF3h were aligned with the JAMM domain sequence of Rpn11. The HxHx_7_Sx_2_D motif necessary for DUB activity of the JAMM domain, missing from Rpn8, Cwf6/Spp42, eIF3h, and eIF3g, is highlighted in green. Residues similar among all proteins are in red, and residues similar among some of the proteins are blue.
(0.64 MB TIF)

Figure S2 **Alignments of Rpn13 proteins from** ***H. sapiens*** **,** ***S. pombe*** **, and** ***S. cerevisiae*** **.** *H. sapiens* Rpn13 sequence was aligned with *S. pombe* Rpn1301 (SPBC342.04), *S. pombe* Rpn1302 (SPCC16A11.16c), and *S. cerevisiae* Rpn13p using Multalin. Residues similar among all proteins are in red, and residues similar among some of the proteins are blue.
(0.48 MB TIF)

Figure S3 **Network diagram of protein interactions of the DUB Ubp8.** The diagram was generated as described in Materials and Methods. DUB nodes are red, SAGA components are yellow, and all other nodes are blue.
(0.22 MB TIF)

Figure S4 **Network diagram of protein interactions of the DUBs Otu1 and Ubp2.** The diagrams were generated as described in Materials and Methods. DUB nodes are red, interactors detected as top hits (TSC) in our TAP/LC-MS/MS analysis and described in the literature for Otu1 and Ubp2 homologs [18],[48],[49],[56] are yellow, and all other nodes are blue.
(0.36 MB TIF)

Figure S5 **Network diagram of protein interactions of the DUB Ubp3.** The diagram was generated as described in Materials and Methods. DUB nodes are red, interactors detected as top hits (TSC) in our TAP/LC-MS/MS analysis and described in the literature for Ubp3 homologs [15],[51] are yellow, direct Ubp3 interactions are green, and all other nodes are blue.
(0.75 MB TIF)

Figure S6 **Network diagram of protein interactions of the DUB Ubp1.** The diagram was generated as described in Materials and Methods. DUB nodes are red, direct Ubp1 interactions are green, and all other nodes are blue. The dashed box denotes the SWI/SNF and RSC complex protein cluster discussed in Results.
(0.92 MB TIF)

Figure S7 **Network diagram of protein interactions of the DUB Ubp7.** The diagram was generated as described in Materials and Methods. DUB nodes are red, direct Ubp7 interactions are green, and all other nodes are blue.
(1.17 MB TIF)

Figure S8 **Network diagram of protein interactions of the DUB Ubp12.** The diagram was generated as described in Materials and Methods. DUB nodes are red, direct Ubp12 interactions are green, and all other nodes are blue.
(0.46 MB TIF)

Figure S9 **Network diagram of protein interactions of the DUBs Ubp14, Ubp15, and Ubp16.** The diagrams were generated as described in Materials and Methods. DUB nodes are red, and all other nodes are blue.
(0.40 MB TIF)

Figure S10 **Network diagram of protein interactions of the DUB Otu2.** The diagram was generated as described in Figure 5 and Materials and Methods. DUB nodes are red, direct Otu2 interactions are green, and all other nodes are blue.
(1.01 MB TIF)

Figure S11 **Network diagram of protein interactions of the DUBs Uch1 and Sst2.** The diagrams were generated as described in Figure 5 and Materials and Methods. DUB nodes are red, and all other nodes are blue.
(0.30 MB TIF)

Figure S12 **Co-localization of Ubp15 and Pob1 at septa.** Cells producing Ubp15 and Pob1 endogenously tagged at their C-termini with mCherry or GFP were imaged by confocal microscopy. Bar: 5 µm.
(0.95 MB TIF)

Figure S13 **Validation of new protein interactions.** (A) Co-IP of Ubp4-GFP and Sfp47-FLAG from cell lysates. Anti-GFP (left side of panels) and anti-FLAG (right side of panels) immunoprecipitates from the indicated strains were blotted with anti-FLAG (top panels) and anti-GFP (bottom panels) antibodies. Asterisks indicate the bands corresponding to Sfp47-FLAG. (B) Co-IP of Ubp5-GFP and Ftp105-V5. Anti-GFP (left side of panels) and anti-V5 (right side of panels) immunoprecipitates from the indicated strains were blotted with anti-V5 (top panels) and anti-GFP (bottom panels) antibodies. (C) Co-IP of TAP-Ubp11 and Tom70-GFP. Anti-GFP (left side of panel) and IgG (right side of panel) immunoprecipitates from the indicated strains were blotted with anti-GFP. (D) Co-IPs among the Ubp9 putative complex components. Anti-GFP (left side of panels) and anti-V5 or IgG (right side of panels) immunoprecipitates from the indicated strains were blotted with anti-GFP (top panels) and anti-V5 or IgG (bottom panels) antibodies. Asterisks indicate the bands corresponding to Ubp9-GFP.
(0.84 MB TIF)

Figure S14 **Ubp4, Ubp5, and Ubp9 expression and modification in different genetic backgrounds.** (A–C) Equivalent amounts of cells expressing Ubp4-GFP, Sfp47-GFP, Ubp5-GFP, Ftp105-GFP, Ubp9-GFP, Bun107-GFP, or Bun62-GFP in the indicated genetic backgrounds were lysed under denaturing conditions. The GFP-tagged proteins were detected by IP followed by immunoblotting. (D) Equivalent amounts of Ubp9-TAP immunoprecipitates were subjected either to lambda phosphatase treatment or a buffer control prior to immunoblotting.
(0.26 MB TIF)

Figure S15 **Characterization of Ubp9 function in actin dynamics, cell polarity, and endocytosis.** (A) Ten-fold dilution series of cells grown to mid-log phase were spotted on YE agar and grown at the indicated temperatures for 3 d. (B) Ten-fold dilution series of cells grown to mid-log phase were spotted on YE agar +/– 2 µM Latrunculin B and grown at 29°C for 3 d. (C) Cells of the indicated genotypes grown to early log phase at 29°C and then shifted to 36°C for 3 h, were labeled with FM4-64 for 10 min and imaged by confocal microscopy. Arrows indicate endocytic vesicles labeled with FM4-64 in wild-type and *myo1Δ* cells. Bar: 5 µm.
(2.96 MB TIF)

Figure S16 **Growth rates and accumulation of ubiquitinated proteins in cells containing multiple DUB deletions.** (A and B) Ten-fold dilution series of cells grown to mid-log phase were spotted on YE agar and grown at the indicated temperatures for 3 d. (C) Anti-ubiquitin immunoblot and Coomassie staining of wild-type or multiple DUB mutant cell lysates produced under fully denaturing conditions.
(3.74 MB TIF)

Figure S17 ***S. cerevisiae*** **Doa4p and** ***H. sapiens*** **USP8 contain an extended N-terminus absent from** ***S. pombe*** **Ubp4.** *S. pombe* Ubp4, *S. cerevisiae* Doa4p, and *H. sapiens* USP8 protein sequences were aligned using Multalin. The MIT (microtubule interacting and transport) domain of USP8 is green. The four motifs necessary for Doa4p targeting to endosomes are yellow.
(0.86 MB TIF)

Figure S18 **Human C17orf28 and fission yeast Ftp105 are homologs.** *H. sapiens* C17orf28 and *S. pombe* Ftp105 sequences were aligned using Multalin. Domain architecture was retrieved using the SMART and Pfam databases.
(0.54 MB TIF)

Table S1 **Proteins recovered from TAPs followed by LC-MS/MS.**
The baits used for the purifications are shown in bold, and shading is as denoted at the end of the table (ubp16 tab). Gene, GeneDB accession number; Seq. cov., protein sequence coverage percent; Uniq. Seq., number of unique peptides identified.
(0.48 MB XLS)

Table S2 **Results from reciprocal TAPs and LC-MS/MS analysis** **for Ubp5, Ubp9, and their partners.**
The baits used for the TAPs are shown in bold. Note that the relative spectral counts from Ubp9 (67 kDa), Bun107 (107 kDa), and Bun62 (62 kDa) are concordant with their relative molecular masses.
(0.04 MB XLS)

Table S3 ***S. pombe*** **strains used in this study.**
(0.22 MB DOC)

## Abbreviations

DUB: deubiquitinating enzyme
ER: endoplasmic reticulum
ESCRT: endosomal sorting complex required for transport
FDR: false discovery rate
GFP: green fluorescent protein
HBH: Histidine_6_-Biotin-Histidine_6_
IP: immunoprecipitation
JAMM: JAB1/MPN/Mov34 metalloenzyme
LC-MS/MS: liquid chromatography–tandem mass spectrometry
MS: mass spectrometry
OTU: ovarian tumor protease
TAP: tandem affinity purification
TSC: total spectral counts
Ub-AMC: ubiquitin 7-amido-4-methylcoumarin
UCH: ubiquitin C-terminal hydrolase
USP: ubiquitin-specific protease
WD repeat: tryptophan–aspartate repeat
YE: yeast extract
