# Supplementary figures and images for: A Global Census of Fission Yeast Deubiquitinating Enzyme Localization and Interaction Networks Reveals Distinct Compartmentalization Profiles and Overlapping Functions in Endocytosis and Polarity
Source: PLoS Biol. 2010 Sep 7;8(9):e1000471. doi: 10.1371/journal.pbio.1000471 (PMC2935449; doi:10.1371/journal.pbio.1000471)

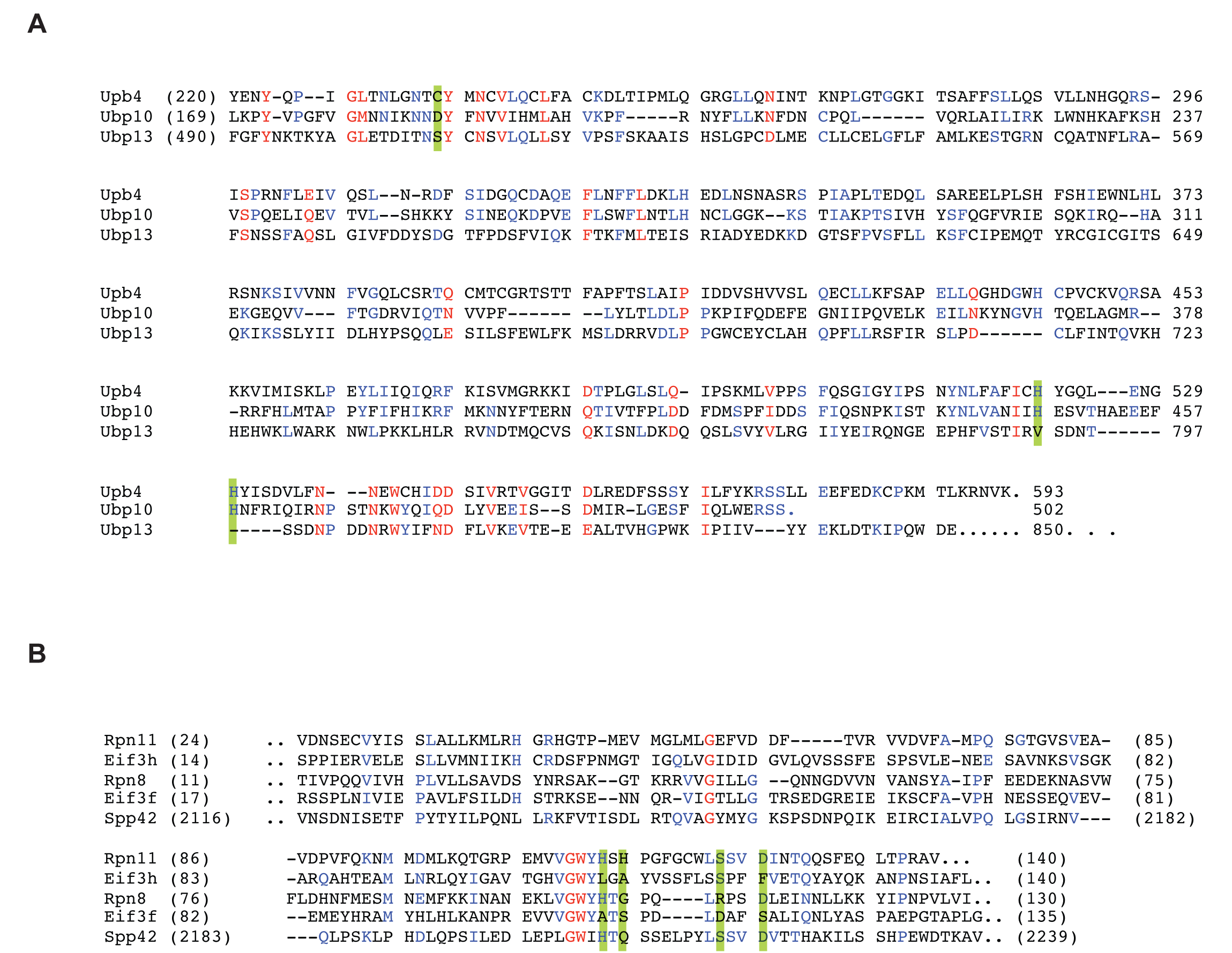

Supplement: Figure S1 — Alignment of S. pombe proteins excluded from our study. (A) The USP domain sequences of Ubp10 and Ubp13 were aligned with the USP domain of Ubp4. The catalytic residues in the Cys and His boxes, respectively, are highlighted in green. Ubp10 lacks the catalytic cysteine but has an intact histidine box. Ubp13 lacks both catalytic boxes. (B) The JAMM domain sequences of Rpn8, Spp42, eIF3f, and eIF3h were aligned with the JAMM domain sequence of Rpn11. The HxHx7Sx2D motif necessary for DUB activity of the JAMM domain, missing from Rpn8, Cwf6/Spp42, eIF3h, and eIF3g, is highlighted in green. Residues similar among all proteins are in red, and residues similar among some of the proteins are blue. (0.64 MB TIF) [file pbio.1000471.s001.tif]

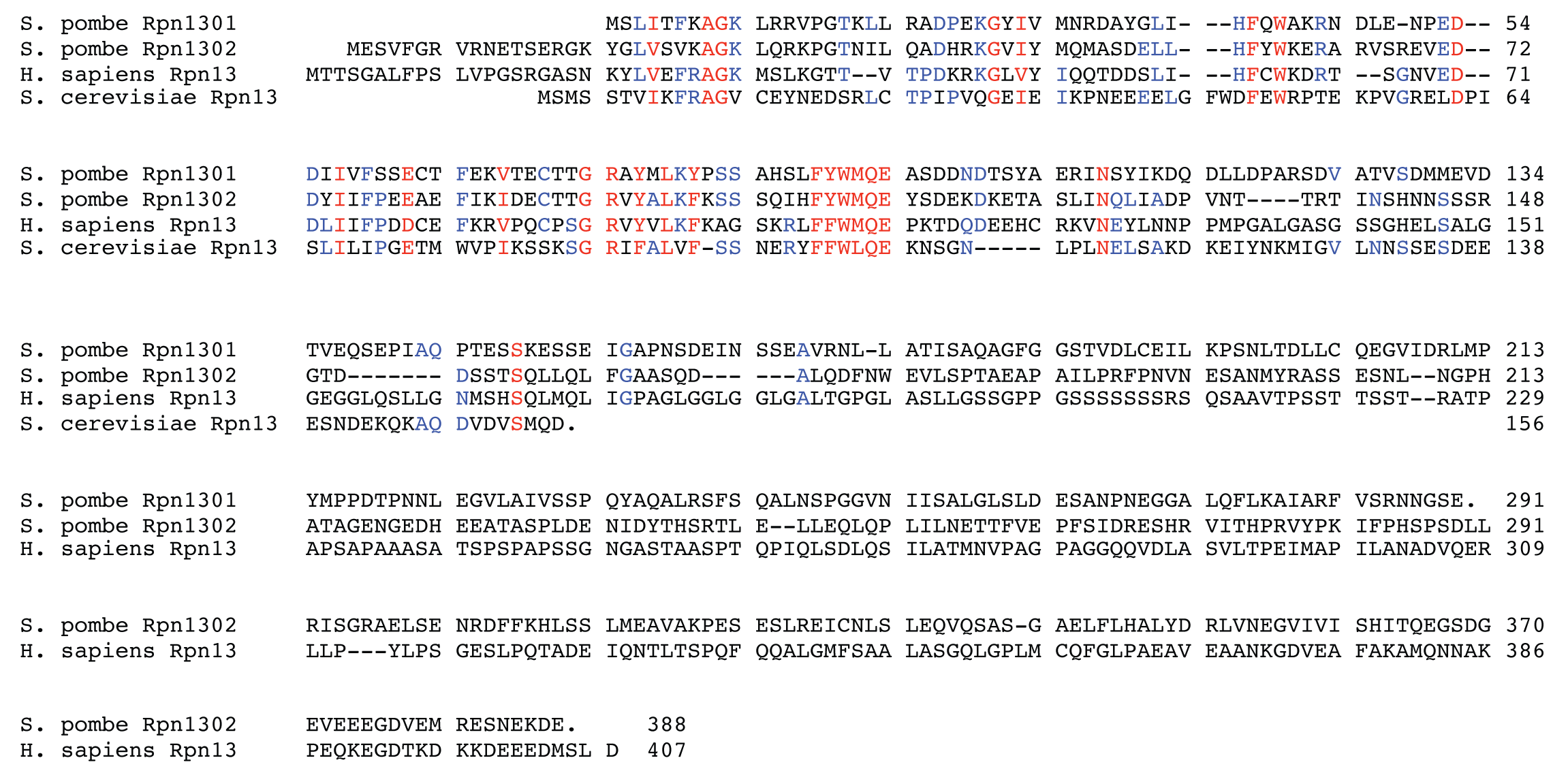

Supplement: Figure S2 — Alignments of Rpn13 proteins from H. sapiens , S. pombe , and S. cerevisiae . H. sapiens Rpn13 sequence was aligned with S. pombe Rpn1301 (SPBC342.04), S. pombe Rpn1302 (SPCC16A11.16c), and S. cerevisiae Rpn13p using Multalin. Residues similar among all proteins are in red, and residues similar among some of the proteins are blue. (0.48 MB TIF) [file pbio.1000471.s002.tif]

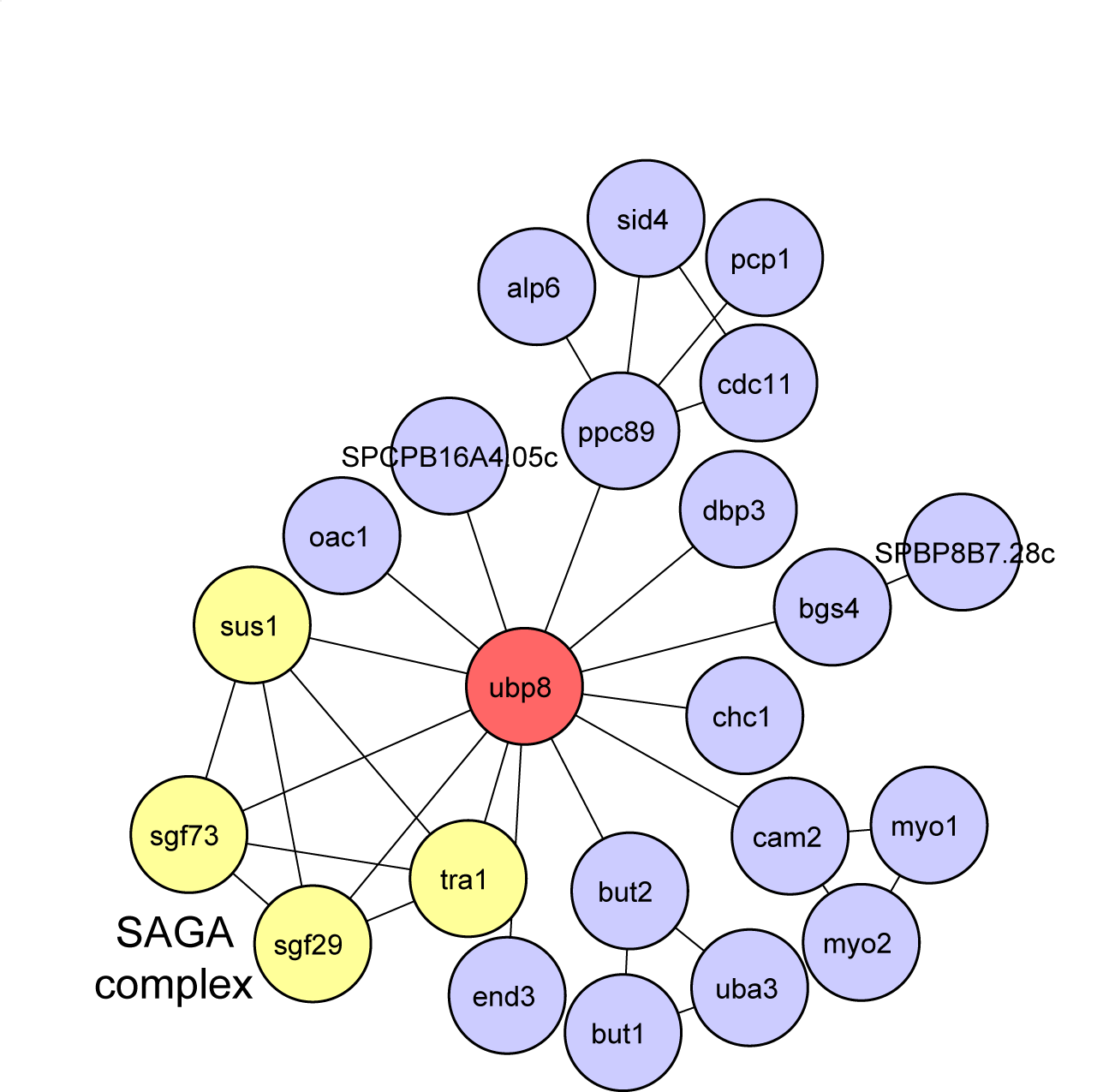

Supplement: Figure S3 — Network diagram of protein interactions of the DUB Ubp8. The diagram was generated as described in Materials and Methods. DUB nodes are red, SAGA components are yellow, and all other nodes are blue. (0.22 MB TIF) [file pbio.1000471.s003.tif]

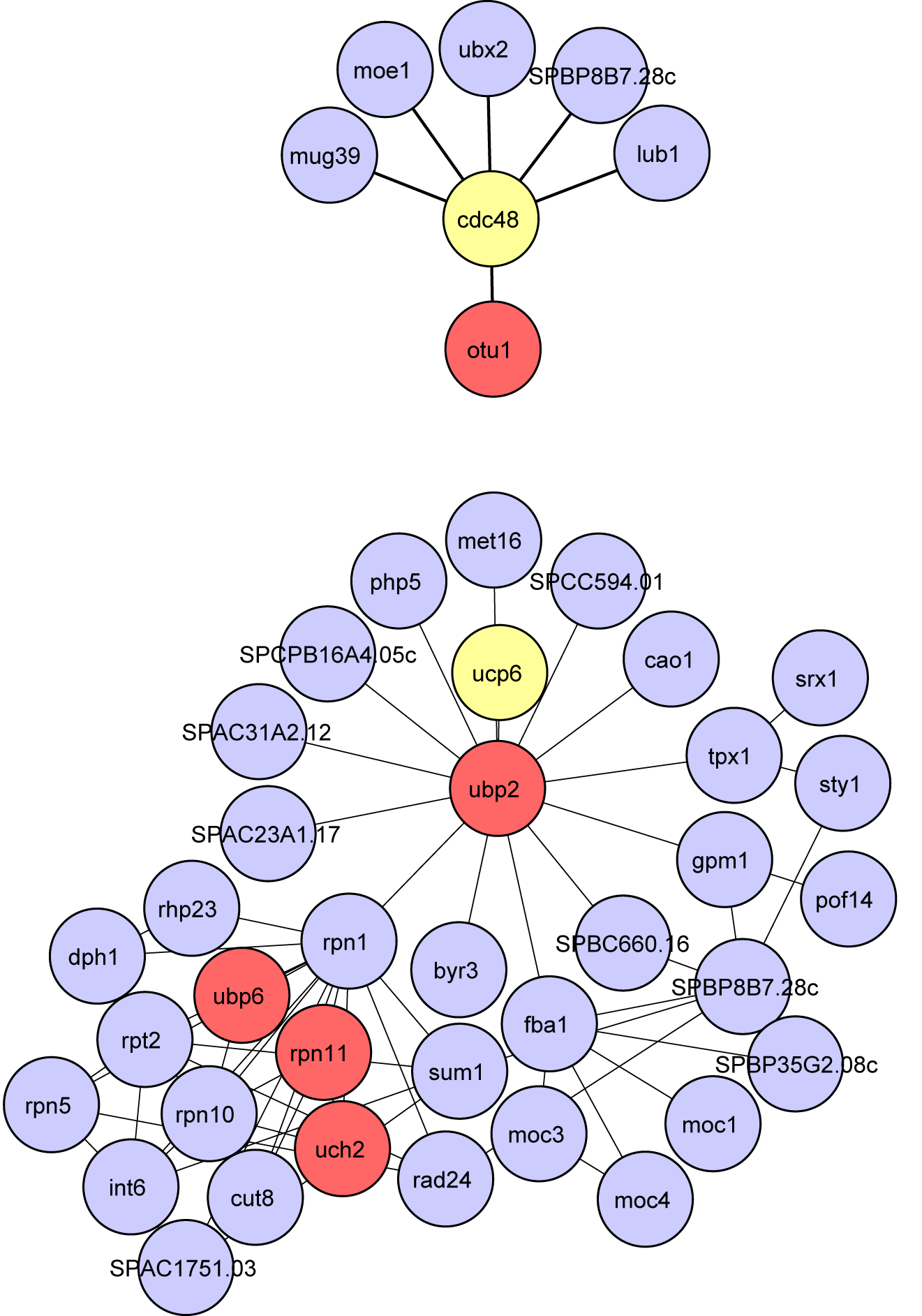

Supplement: Figure S4 — Network diagram of protein interactions of the DUBs Otu1 and Ubp2. The diagrams were generated as described in Materials and Methods. DUB nodes are red, interactors detected as top hits (TSC) in our TAP/LC-MS/MS analysis and described in the literature for Otu1 and Ubp2 homologs [18],[48],[49],[56] are yellow, and all other nodes are blue. (0.36 MB TIF) [file pbio.1000471.s004.tif]

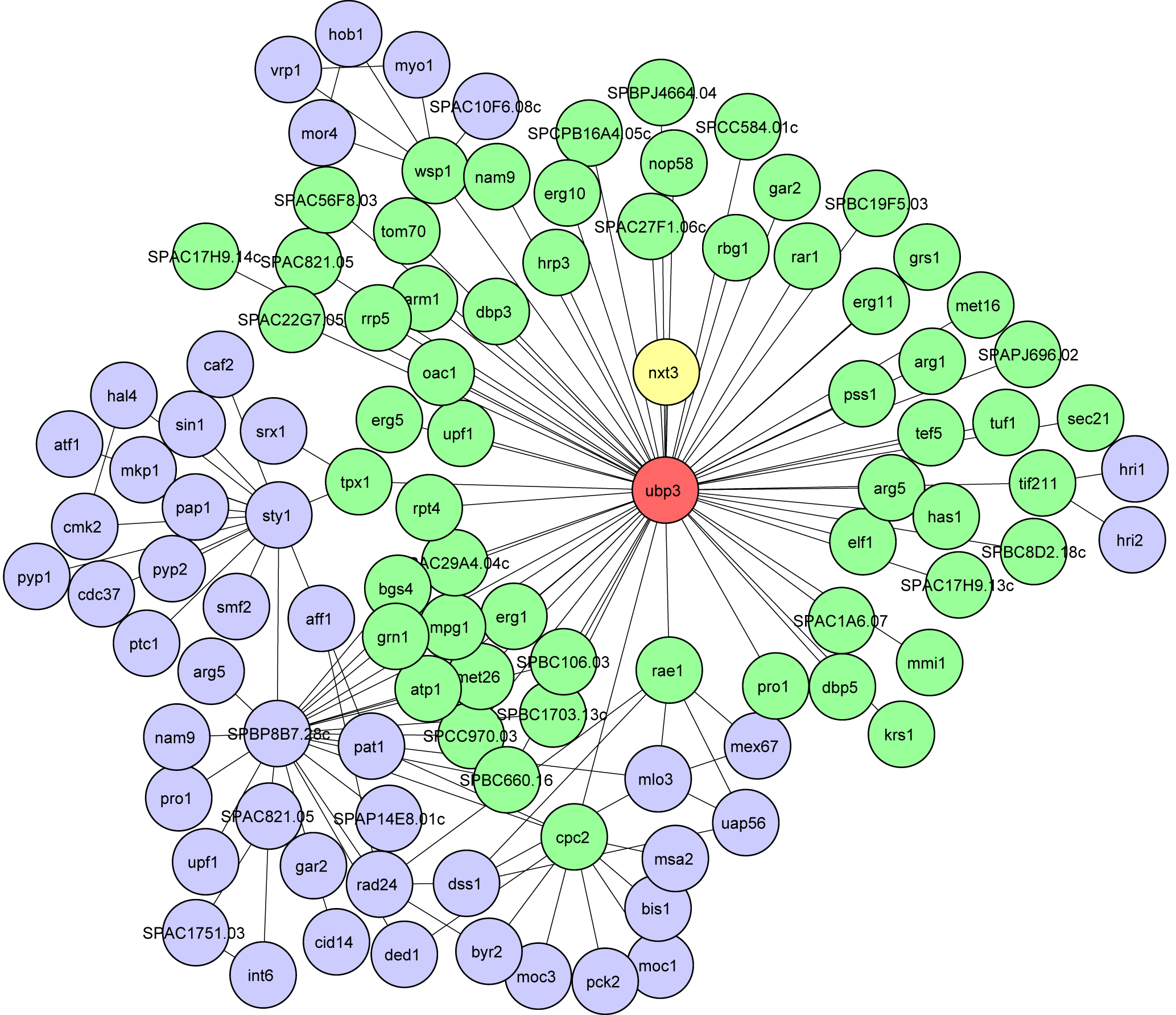

Supplement: Figure S5 — Network diagram of protein interactions of the DUB Ubp3. The diagram was generated as described in Materials and Methods. DUB nodes are red, interactors detected as top hits (TSC) in our TAP/LC-MS/MS analysis and described in the literature for Ubp3 homologs [15],[51] are yellow, direct Ubp3 interactions are green, and all other nodes are blue. (0.75 MB TIF) [file pbio.1000471.s005.tif]

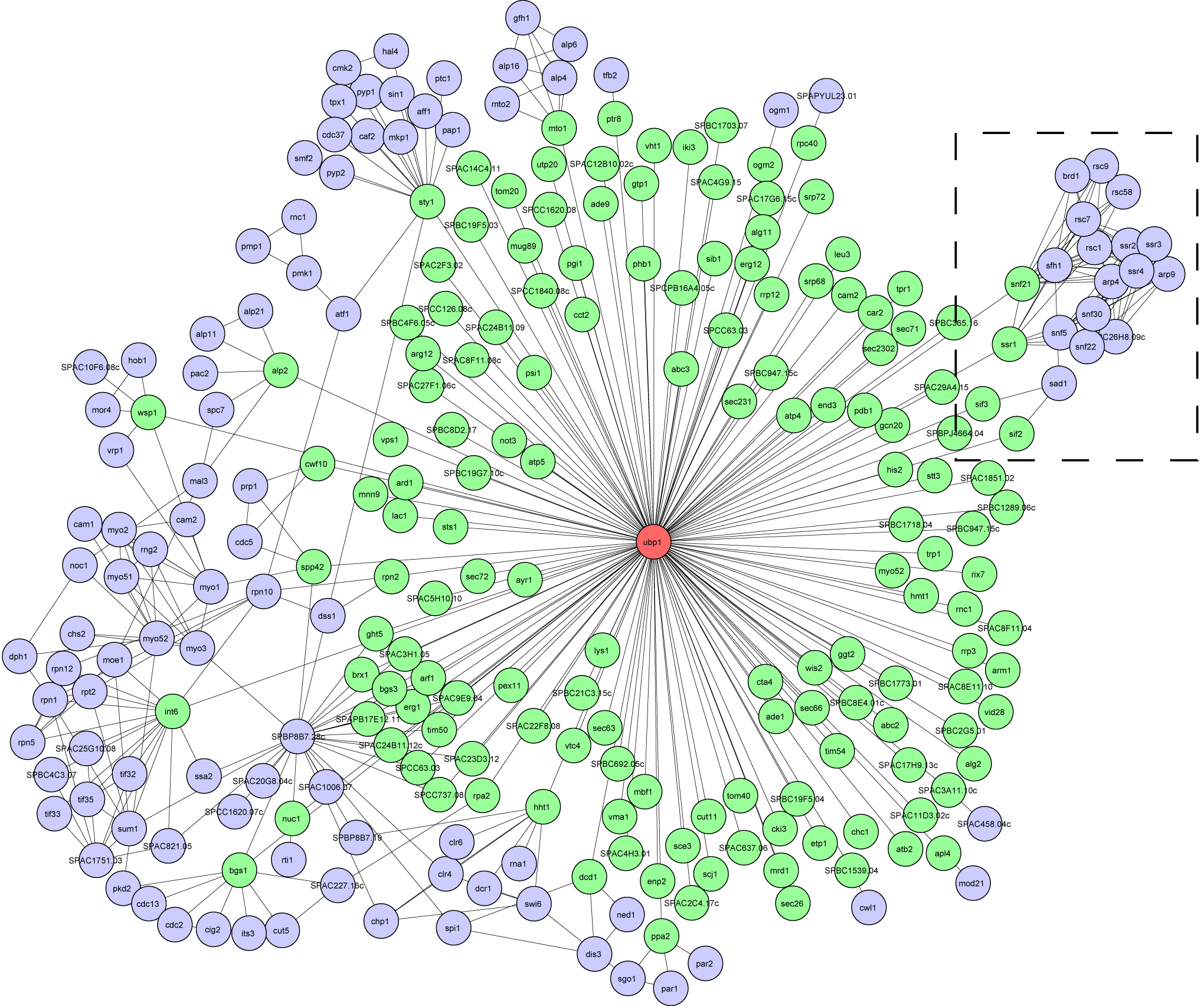

Supplement: Figure S6 — Network diagram of protein interactions of the DUB Ubp1. The diagram was generated as described in Materials and Methods. DUB nodes are red, direct Ubp1 interactions are green, and all other nodes are blue. The dashed box denotes the SWI/SNF and RSC complex protein cluster discussed in Results. (0.92 MB TIF) [file pbio.1000471.s006.tif]

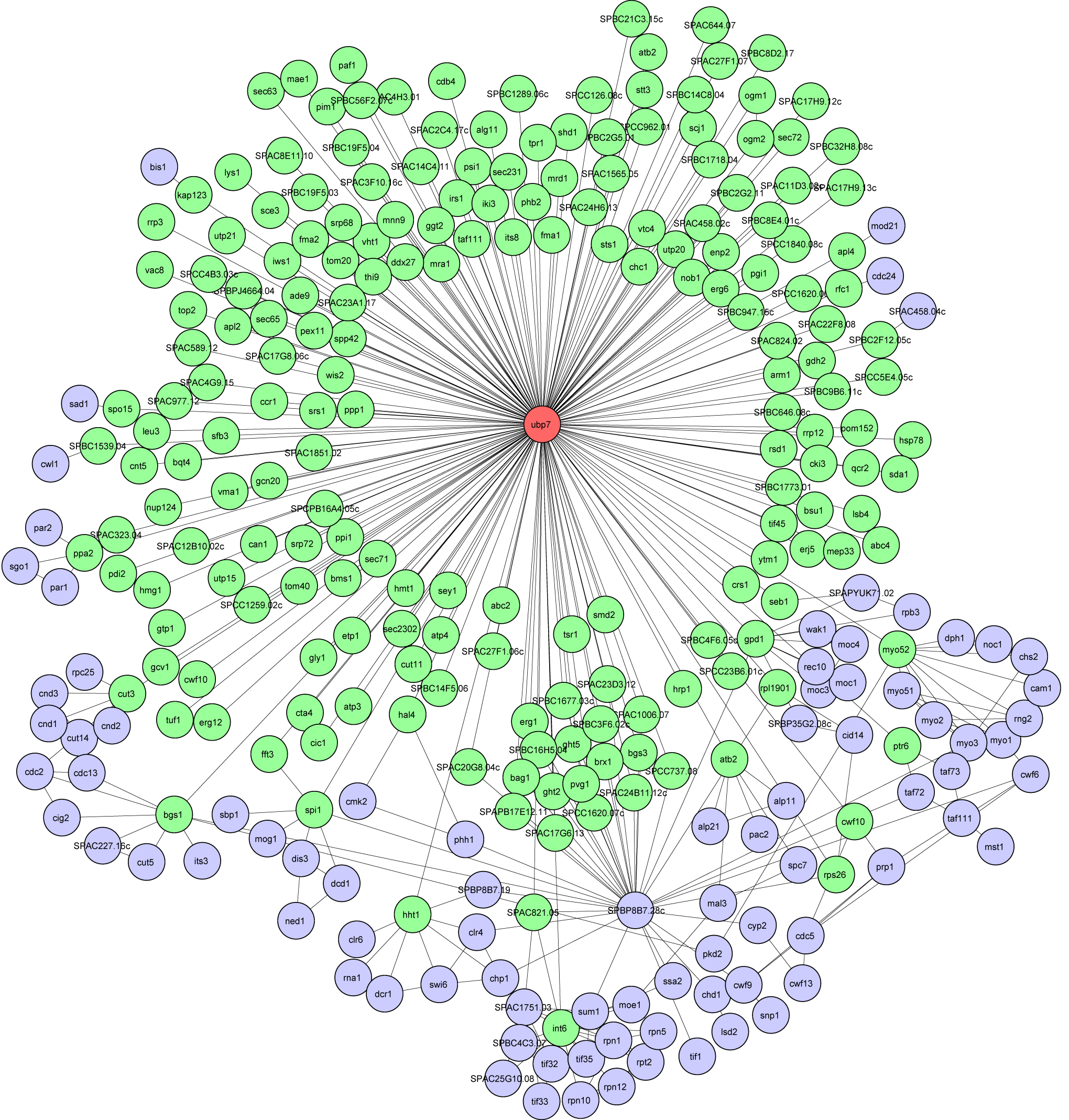

Supplement: Figure S7 — Network diagram of protein interactions of the DUB Ubp7. The diagram was generated as described in Materials and Methods. DUB nodes are red, direct Ubp7 interactions are green, and all other nodes are blue. (1.17 MB TIF) [file pbio.1000471.s007.tif]

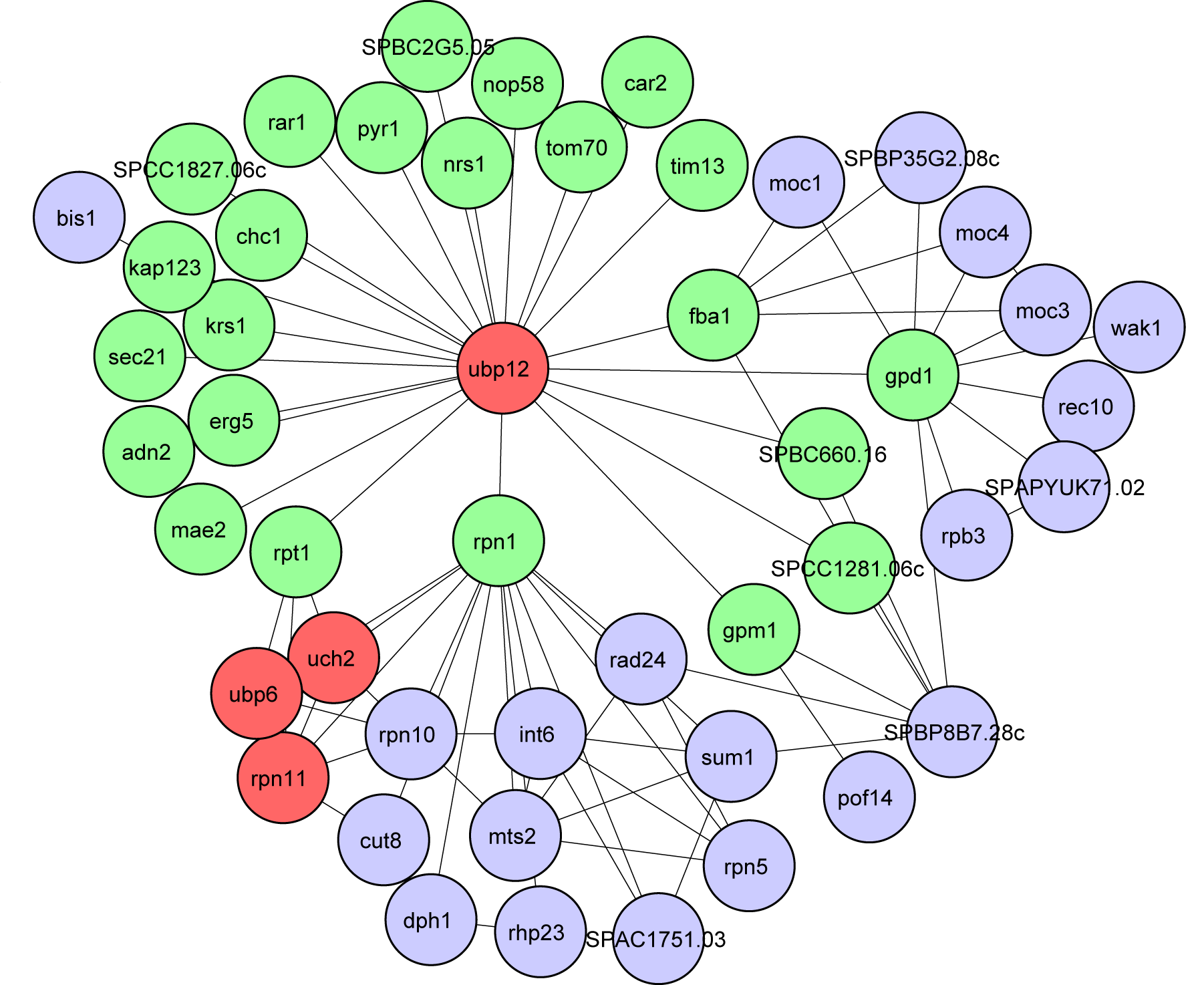

Supplement: Figure S8 — Network diagram of protein interactions of the DUB Ubp12. The diagram was generated as described in Materials and Methods. DUB nodes are red, direct Ubp12 interactions are green, and all other nodes are blue. (0.46 MB TIF) [file pbio.1000471.s008.tif]

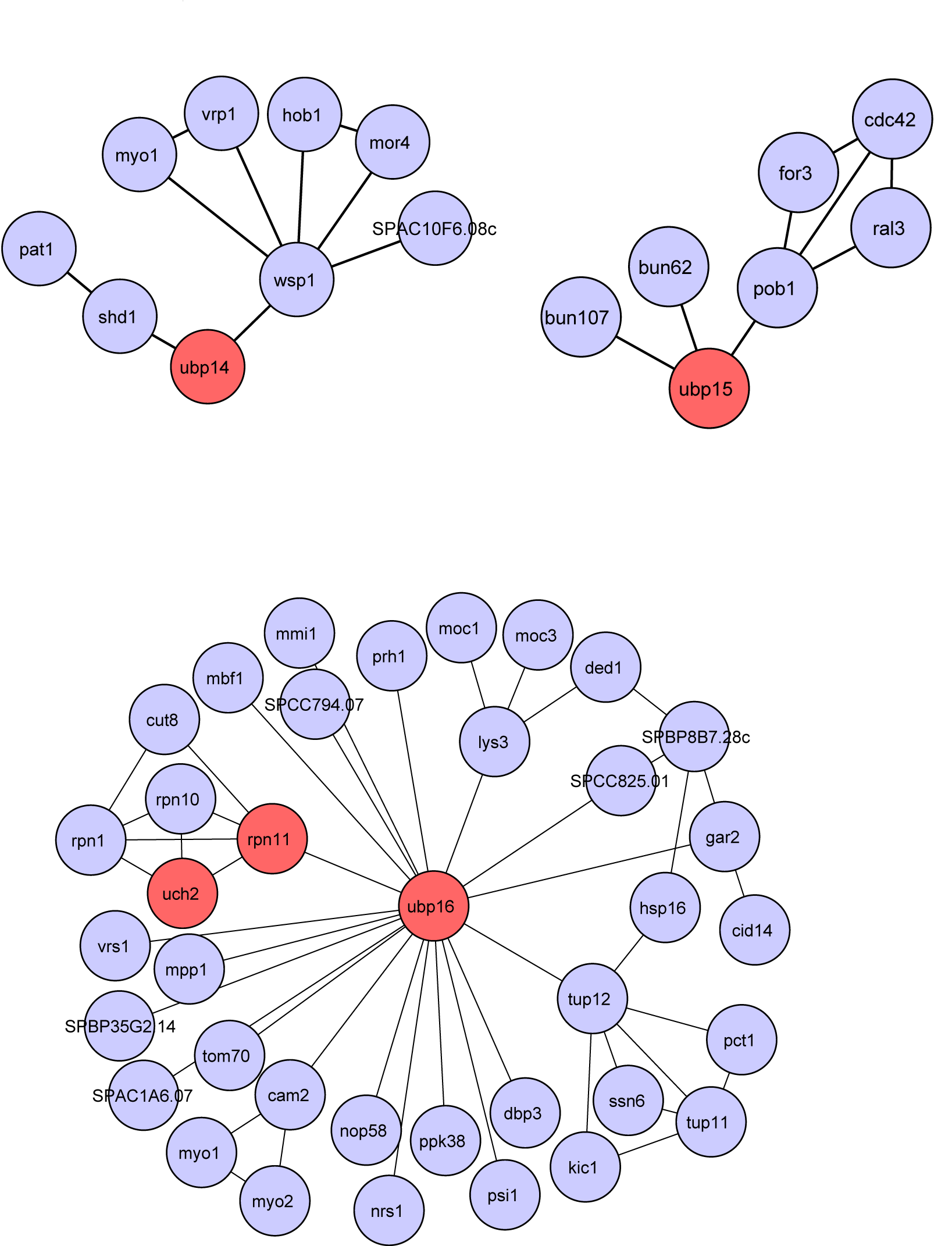

Supplement: Figure S9 — Network diagram of protein interactions of the DUBs Ubp14, Ubp15, and Ubp16. The diagrams were generated as described in Materials and Methods. DUB nodes are red, and all other nodes are blue. (0.40 MB TIF) [file pbio.1000471.s009.tif]

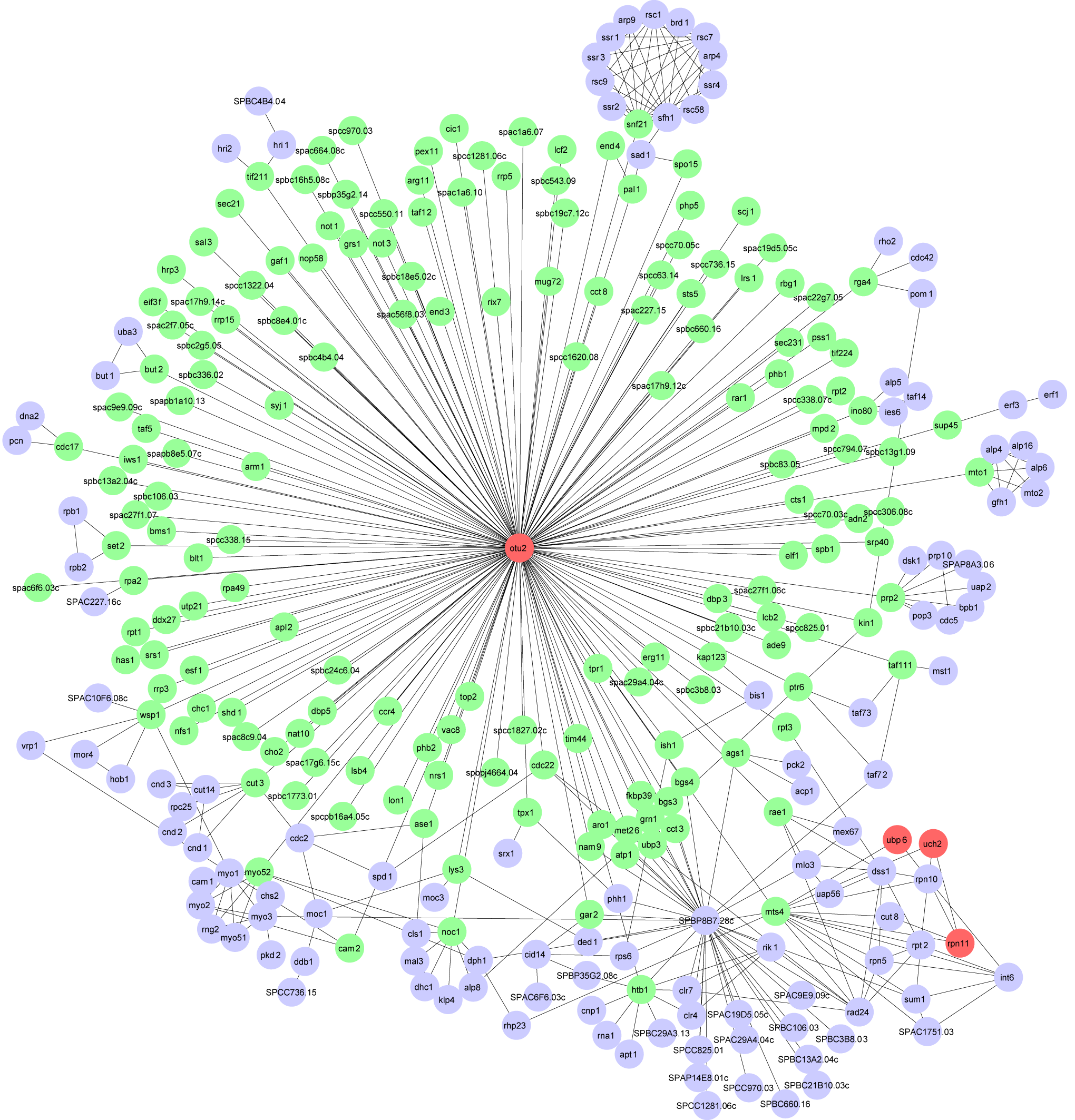

Supplement: Figure S10 — Network diagram of protein interactions of the DUB Otu2. The diagram was generated as described in Figure 5 and Materials and Methods. DUB nodes are red, direct Otu2 interactions are green, and all other nodes are blue. (1.01 MB TIF) [file pbio.1000471.s010.tif]

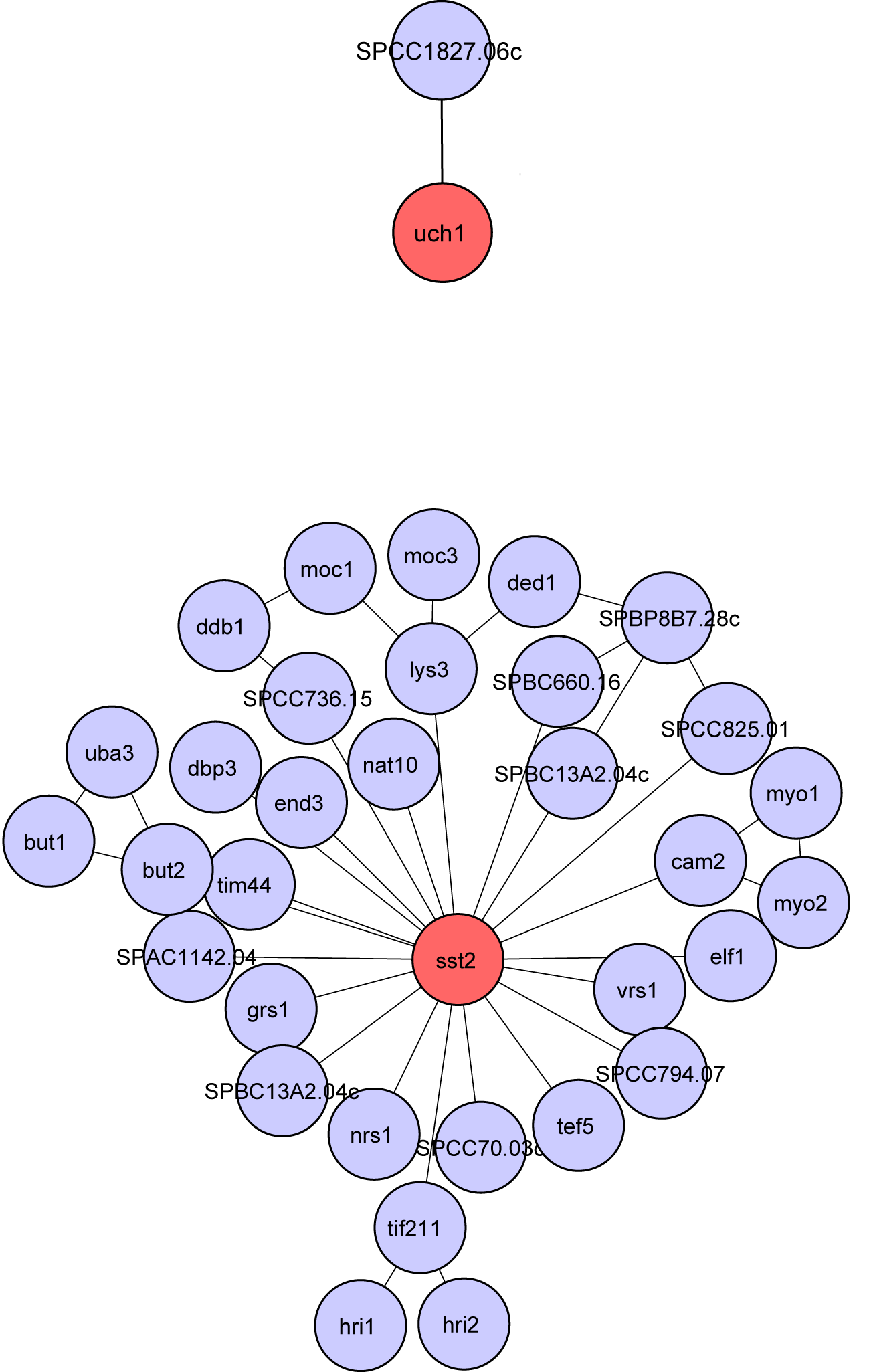

Supplement: Figure S11 — Network diagram of protein interactions of the DUBs Uch1 and Sst2. The diagrams were generated as described in Figure 5 and Materials and Methods. DUB nodes are red, and all other nodes are blue. (0.30 MB TIF) [file pbio.1000471.s011.tif]

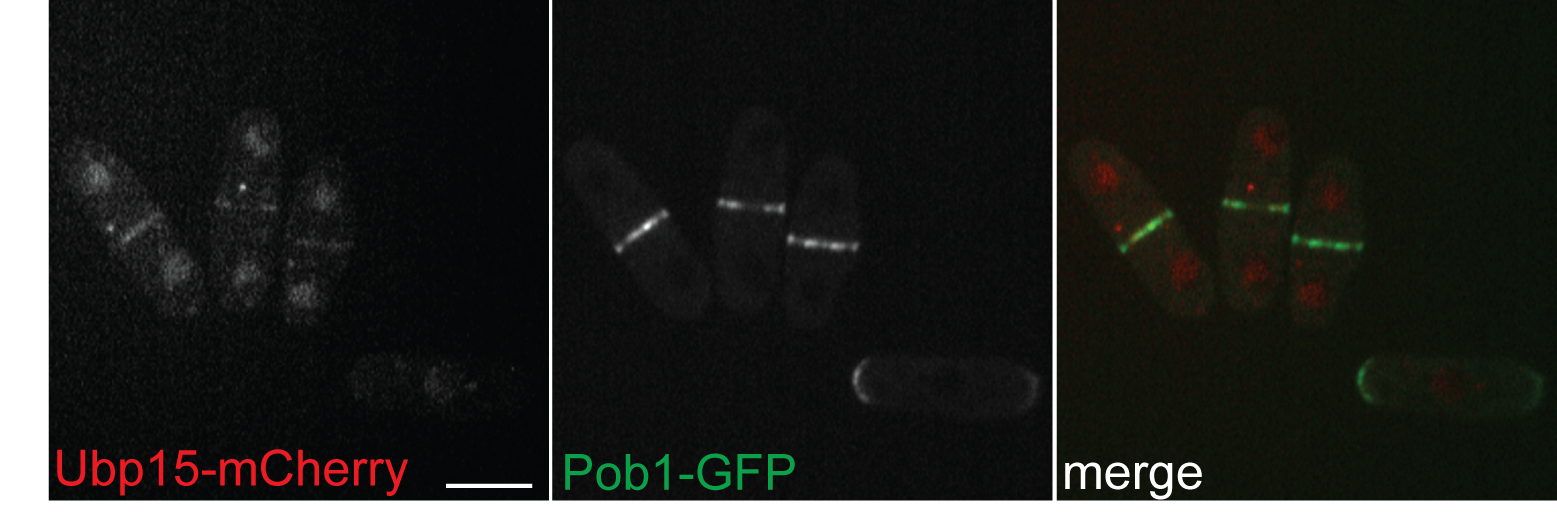

Supplement: Figure S12 — Co-localization of Ubp15 and Pob1 at septa. Cells producing Ubp15 and Pob1 endogenously tagged at their C-termini with mCherry or GFP were imaged by confocal microscopy. Bar: 5 µm. (0.95 MB TIF) [file pbio.1000471.s012.tif]

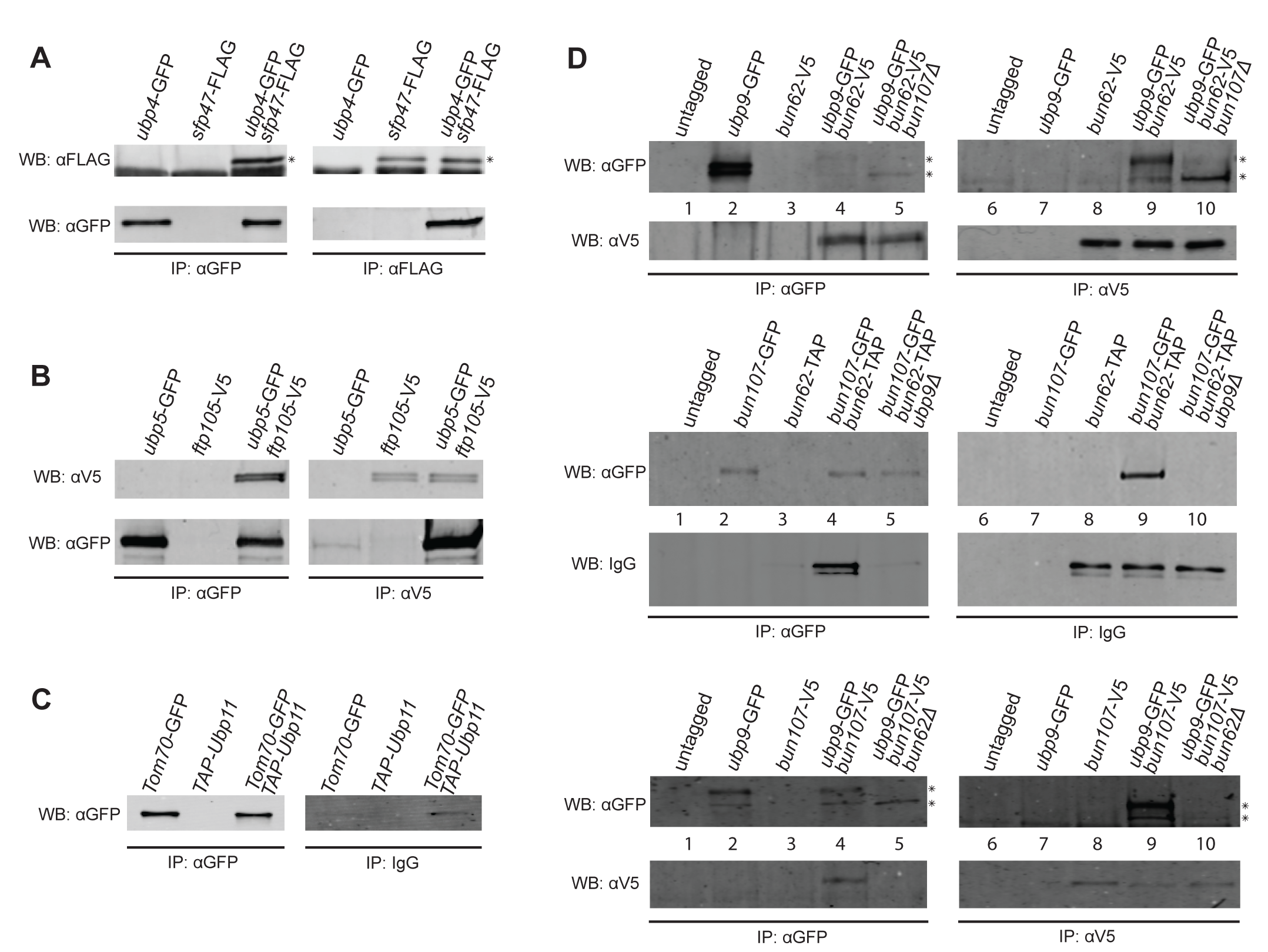

Supplement: Figure S13 — Validation of new protein interactions. (A) Co-IP of Ubp4-GFP and Sfp47-FLAG from cell lysates. Anti-GFP (left side of panels) and anti-FLAG (right side of panels) immunoprecipitates from the indicated strains were blotted with anti-FLAG (top panels) and anti-GFP (bottom panels) antibodies. Asterisks indicate the bands corresponding to Sfp47-FLAG. (B) Co-IP of Ubp5-GFP and Ftp105-V5. Anti-GFP (left side of panels) and anti-V5 (right side of panels) immunoprecipitates from the indicated strains were blotted with anti-V5 (top panels) and anti-GFP (bottom panels) antibodies. (C) Co-IP of TAP-Ubp11 and Tom70-GFP. Anti-GFP (left side of panel) and IgG (right side of panel) immunoprecipitates from the indicated strains were blotted with anti-GFP. (D) Co-IPs among the Ubp9 putative complex components. Anti-GFP (left side of panels) and anti-V5 or IgG (right side of panels) immunoprecipitates from the indicated strains were blotted with anti-GFP (top panels) and anti-V5 or IgG (bottom panels) antibodies. Asterisks indicate the bands corresponding to Ubp9-GFP. (0.84 MB TIF) [file pbio.1000471.s013.tif]

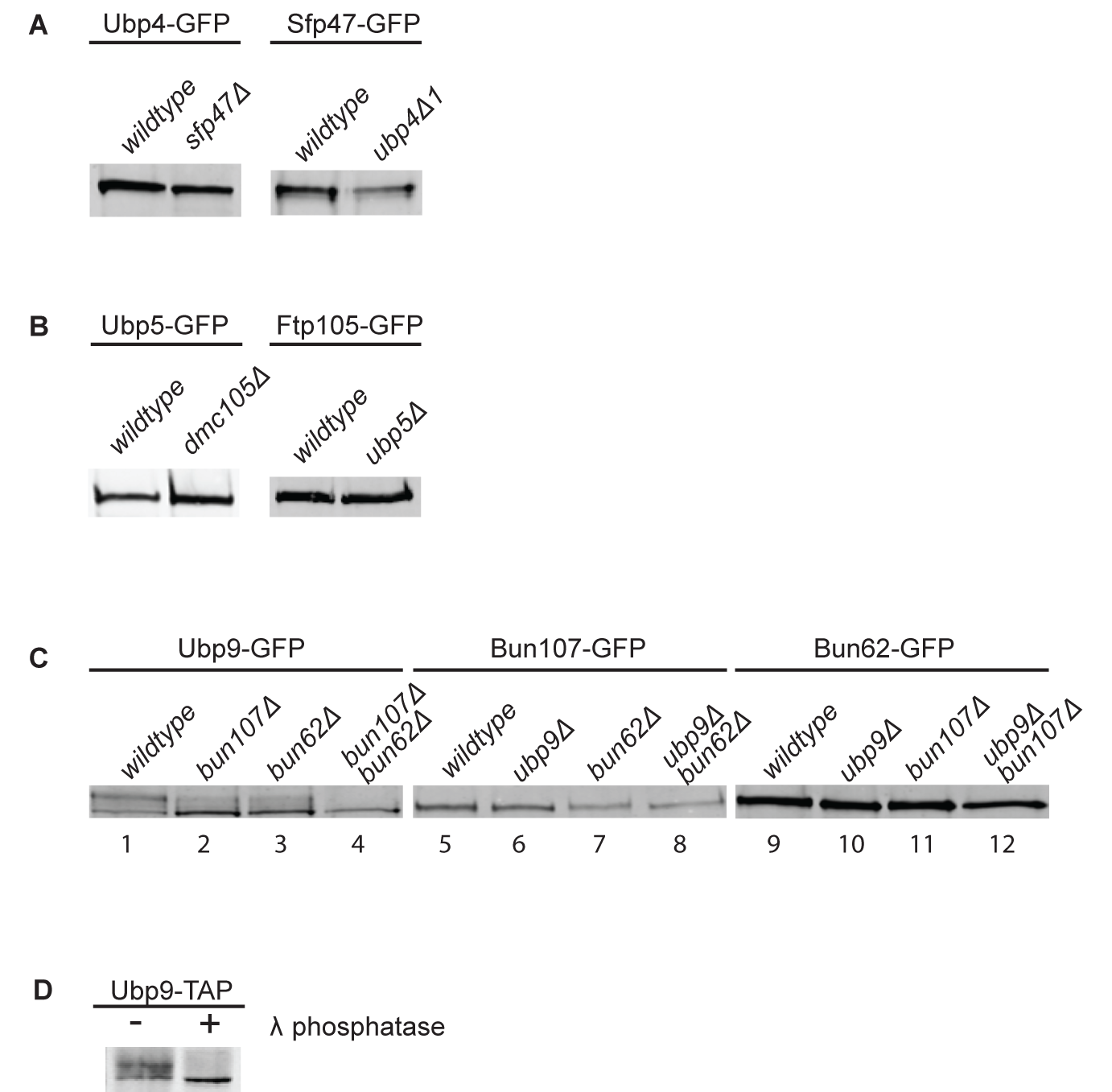

Supplement: Figure S14 — Ubp4, Ubp5, and Ubp9 expression and modification in different genetic backgrounds. (A–C) Equivalent amounts of cells expressing Ubp4-GFP, Sfp47-GFP, Ubp5-GFP, Ftp105-GFP, Ubp9-GFP, Bun107-GFP, or Bun62-GFP in the indicated genetic backgrounds were lysed under denaturing conditions. The GFP-tagged proteins were detected by IP followed by immunoblotting. (D) Equivalent amounts of Ubp9-TAP immunoprecipitates were subjected either to lambda phosphatase treatment or a buffer control prior to immunoblotting. (0.26 MB TIF) [file pbio.1000471.s014.tif]

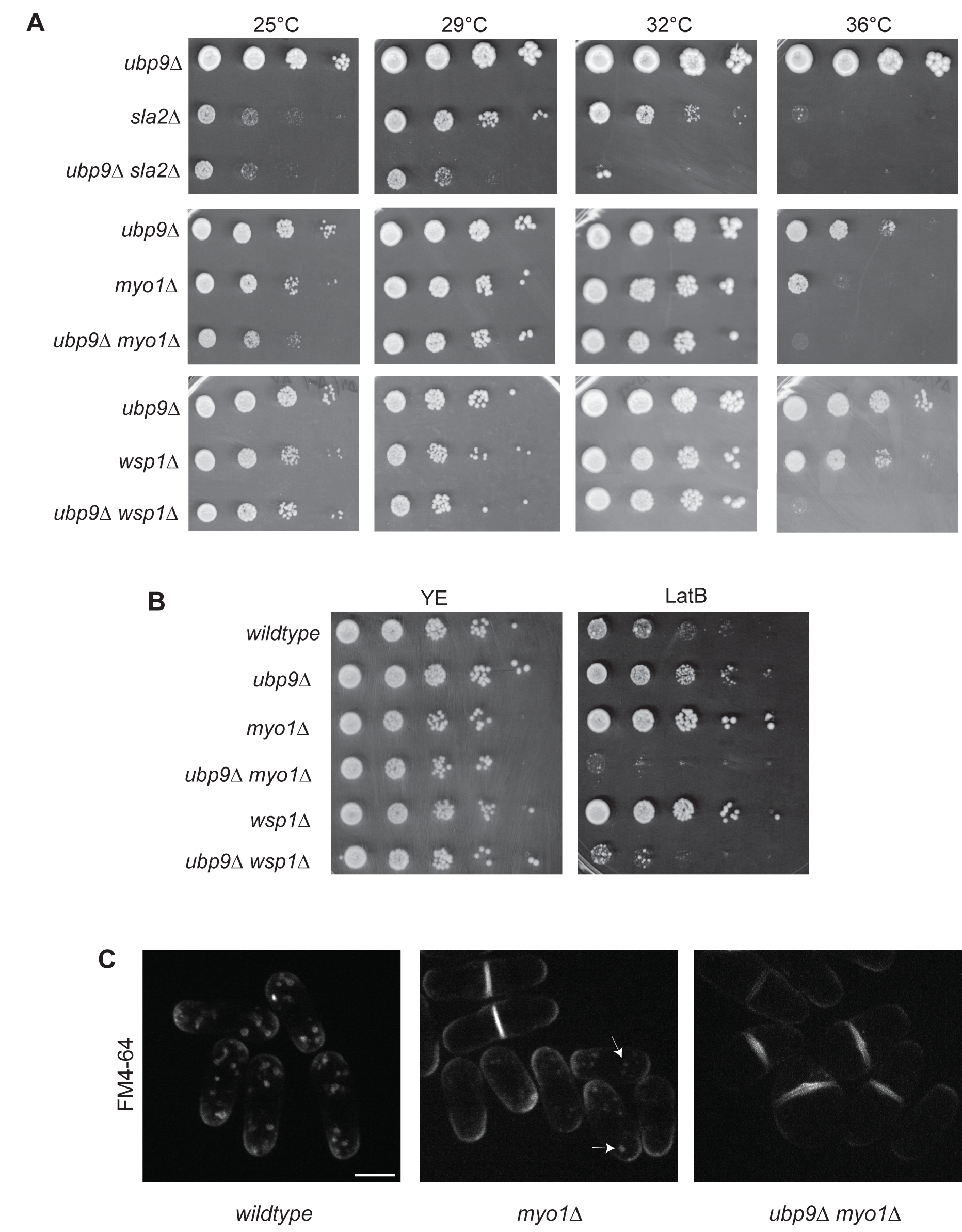

Supplement: Figure S15 — Characterization of Ubp9 function in actin dynamics, cell polarity, and endocytosis. (A) Ten-fold dilution series of cells grown to mid-log phase were spotted on YE agar and grown at the indicated temperatures for 3 d. (B) Ten-fold dilution series of cells grown to mid-log phase were spotted on YE agar +/– 2 µM Latrunculin B and grown at 29°C for 3 d. (C) Cells of the indicated genotypes grown to early log phase at 29°C and then shifted to 36°C for 3 h, were labeled with FM4-64 for 10 min and imaged by confocal microscopy. Arrows indicate endocytic vesicles labeled with FM4-64 in wild-type and myo1Δ cells. Bar: 5 µm. (2.96 MB TIF) [file pbio.1000471.s015.tif]

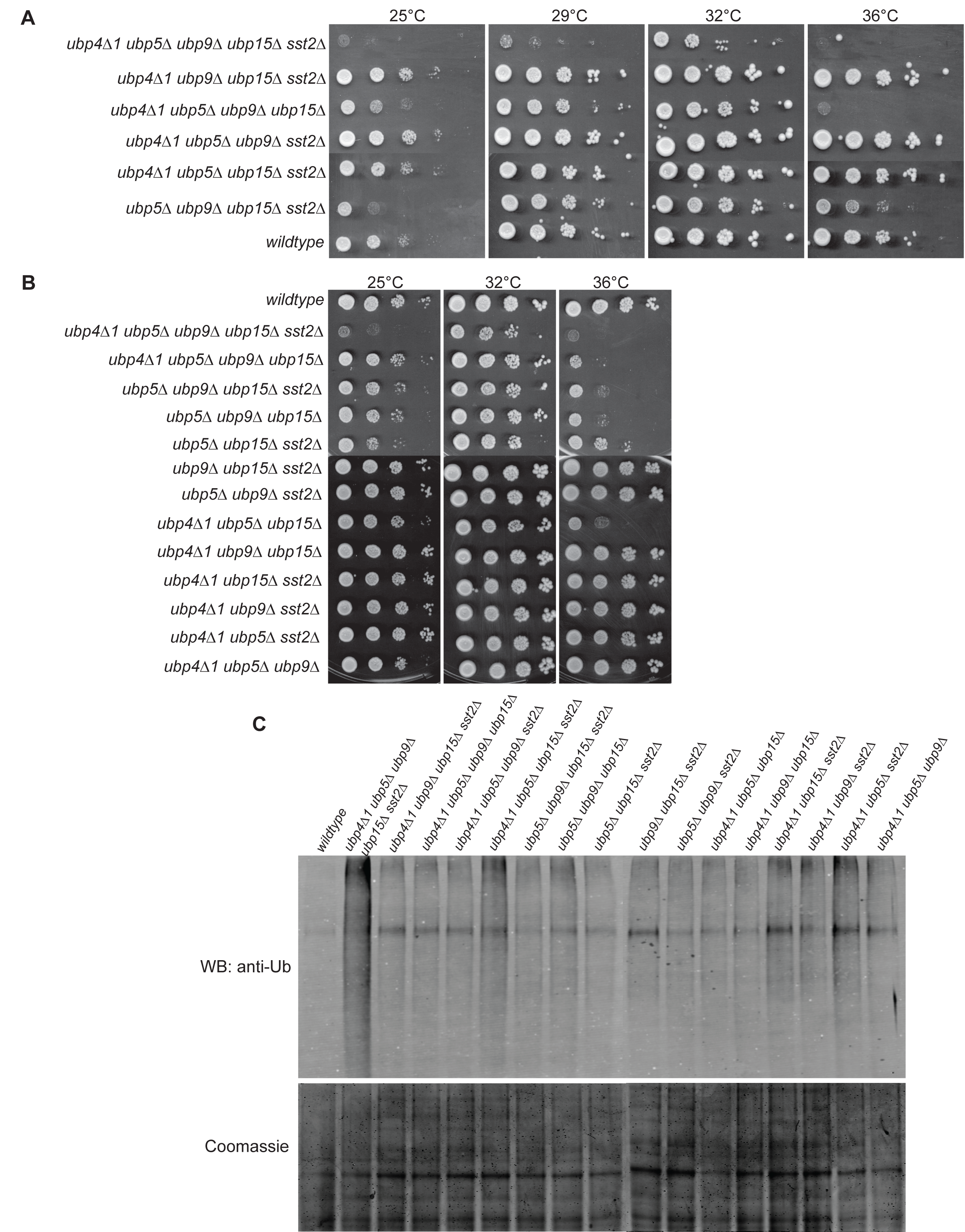

Supplement: Figure S16 — Growth rates and accumulation of ubiquitinated proteins in cells containing multiple DUB deletions. (A and B) Ten-fold dilution series of cells grown to mid-log phase were spotted on YE agar and grown at the indicated temperatures for 3 d. (C) Anti-ubiquitin immunoblot and Coomassie staining of wild-type or multiple DUB mutant cell lysates produced under fully denaturing conditions. (3.74 MB TIF) [file pbio.1000471.s016.tif]

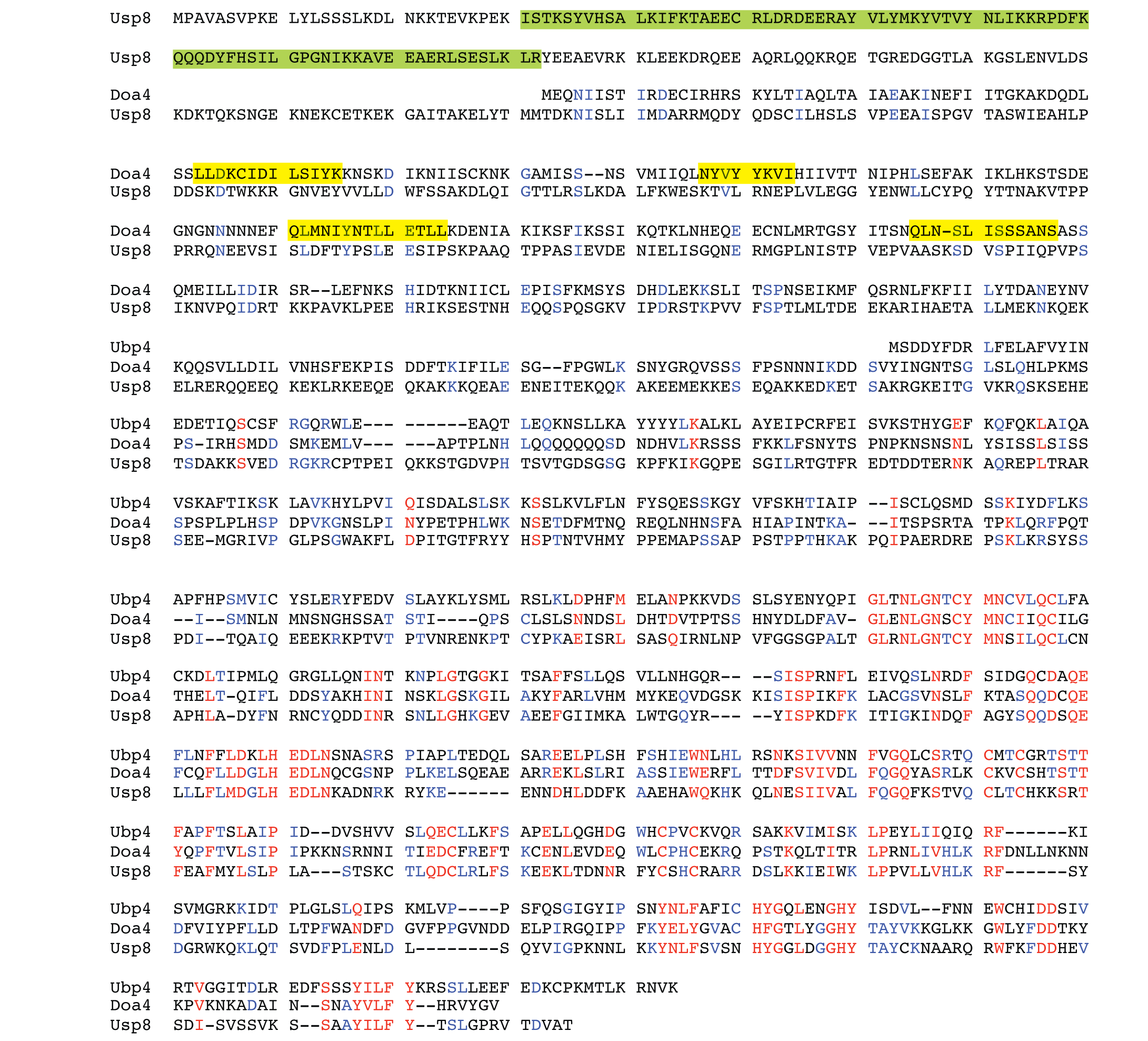

Supplement: Figure S17 — S. cerevisiae Doa4p and H. sapiens USP8 contain an extended N-terminus absent from S. pombe Ubp4. S. pombe Ubp4, S. cerevisiae Doa4p, and H. sapiens USP8 protein sequences were aligned using Multalin. The MIT (microtubule interacting and transport) domain of USP8 is green. The four motifs necessary for Doa4p targeting to endosomes are yellow. (0.86 MB TIF) [file pbio.1000471.s017.tif]

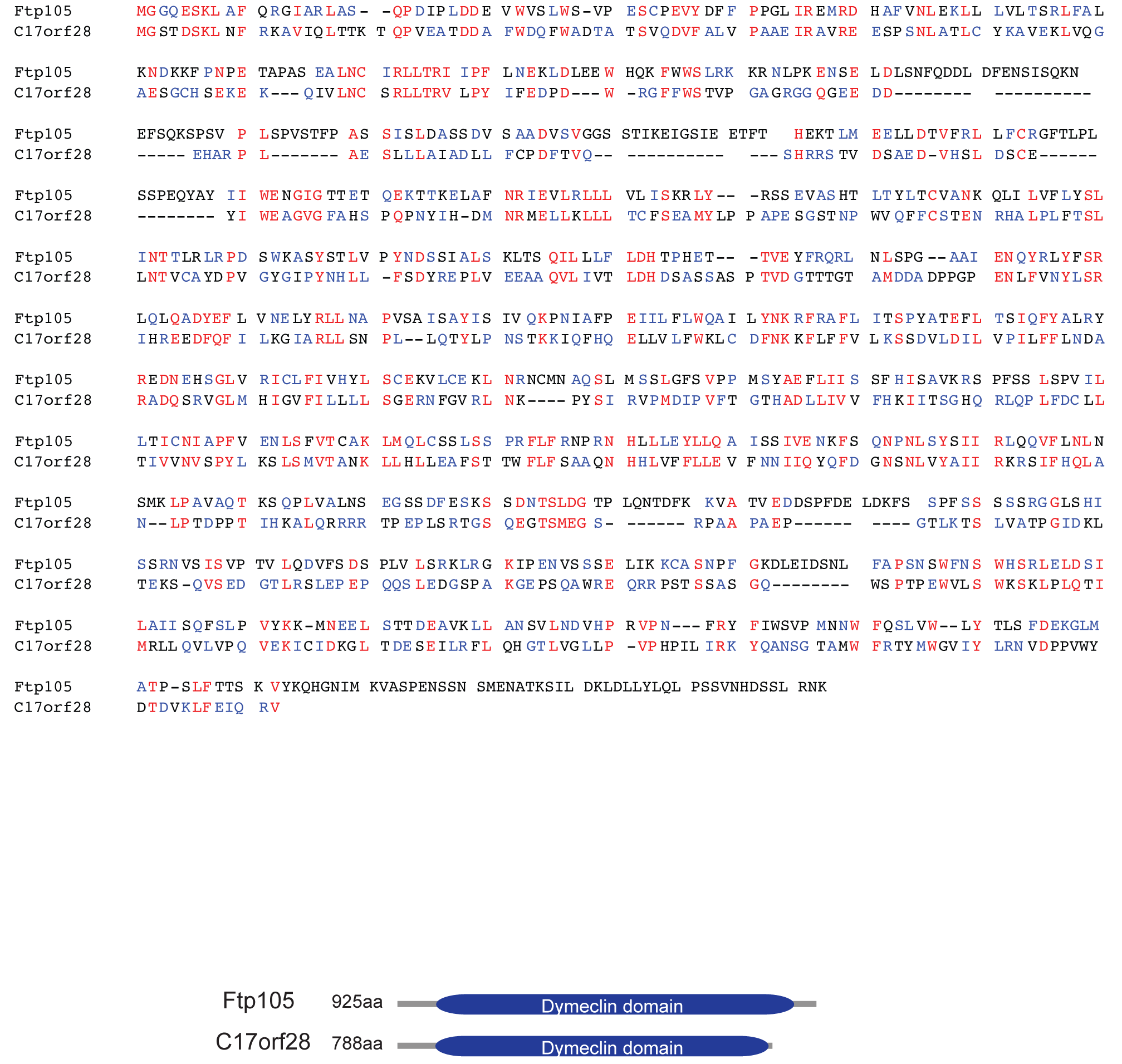

Supplement: Figure S18 — Human C17orf28 and fission yeast Ftp105 are homologs. H. sapiens C17orf28 and S. pombe Ftp105 sequences were aligned using Multalin. Domain architecture was retrieved using the SMART and Pfam databases. (0.54 MB TIF) [file pbio.1000471.s018.tif]
