# Supplementary material for: A Global Census of Fission Yeast Deubiquitinating Enzyme Localization and Interaction Networks Reveals Distinct Compartmentalization Profiles and Overlapping Functions in Endocytosis and Polarity
Source: PLoS Biol. 2010 Sep 7;8(9):e1000471. doi: 10.1371/journal.pbio.1000471 (PMC2935449; doi:10.1371/journal.pbio.1000471)
Supplement: Table S3 — S. pombe strains used in this study. (0.22 MB DOC) [file pbio.1000471.s021.doc]

**Table S3. *S. pombe*** strains used in this study

| **Strain number**  **KGY** | **Genotype** | **Source** |
| --- | --- | --- |
| 246 | *ade6*-M210 *ura4*-D18 *leu1*-32 h- | laboratory stock |
| 586 | *myo1*::kanR *ade6*-M216 *leu1*-32 h- | M. Yamamoto |
| 6303 | *ubp9*-TAP::kanR *ade6*-M210 *ura4*-D18 *leu1*-32 h- | this study |
| 7027 | *bun107*-TAP::kanR *ade6*-M216 *ura4*-D18 *leu1*-32 h+ | this study |
| 7155 | *uch1*-TAP::kanR *ade6*-M210 *ura4*-D18 *leu1*-32 h- | this study |
| 7156 | *uch2*-TAP::kanR *ade6*-M210 *ura4*-D18 *leu1*-32 h- | this study |
| 7157 | *ubp4*-TAP::kanR *ade6*-M210 *ura4*-D18 *leu1*-32 h- | this study |
| 7158 | *ubp7*-TAP::kanR *ade6*-M210 *ura4*-D18 *leu1*-32 h- | this study |
| 7159 | *ubp8*-TAP::kanR *ade6*-M210 *ura4*-D18 *leu1*-32 h- | this study |
| 7162 | *ubp14*-TAP::kanR *ade6*-M210 *ura4*-D18 *leu1*-32 h- | this study |
| 7164 | *ubp2*-TAP::kanR *ade6*-M210 *ura4*-D18 *leu1*-32 h- | this study |
| 7165 | *ubp5*-TAP::kanR *ade6*-M210 *ura4*-D18 *leu1*-32 h- | this study |
| 7166 | *bun62*-TAP::kanR *ade6*-M210 *ura4*-D18 *leu1*-32 h- | this study |
| 7240 | *ubp9::ura4+* *ade6*-M21X *ura4*-D18 *leu1*-32 h+ | this study |
| 7311 | *uch1*-GFP::kanR *ade6*-M210 *ura4*-D18 *leu1*-32 h- | this study |
| 7313 | *ubp4*-GFP::kanR *ade6*-M210 *ura4*-D18 *leu1*-32 h- | this study |
| 7315 | *ubp8*-GFP::kanR *ade6*-M210 *ura4*-D18 *leu1*-32 h- | this study |
| 7319 | *ubp14*-GFP::kanR *ade6*-M210 *ura4*-D18 *leu1*-32 h- | this study |
| 7336 | *ubp6*-GFP::kanR *ade6*-M210 *ura4*-D18 *leu1*-32 h- | this study |
| 7337 | *ubp12*-GFP::kanR *ade6*-M210 *ura4*-D18 *leu1*-32 h- | this study |
| 7339 | *ubp5*-GFP::kanR *ade6*-M210 *ura4*-D18 *leu1*-32 h- | this study |
| 7341 | *bun107*-GFP::kanR *ade6*-M210 *ura4*-D18 *leu1*-32 h- | this study |
| 7352 | *bun107::ura4+ ade6*-M210 *ura4*-D18 *leu1*-32 h- | this study |
| 7353 | *bun62::ura4+ ade6*-M210 *ura4*-D18 *leu1*-32 h- | this study |
| 7415 | *ubp15*-GFP::kanR *ade6*-M210 *ura4*-D18 *leu1*-32 h- | this study |
| 7422 | *ubp9*-GFP::kanR *ade6*-M210 *ura4*-D18 *leu1*-32 h- | this study |
| 7512 | *ubp9*-GFP::kanR *bun107::ura4+* *ade6*-M210 *ura4*-D18 *leu1*-32 h- | this study |
| 7513 | *ubp9*-GFP::kanR *bun62::ura4+* *ade6*-M210 *ura4*-D18 *leu1*-32 h+ | this study |
| 7515 | *bun107*-GFP::kanR *ubp9::ura4+* *ade6*-M21X *ura4*-D18 *leu1*-32 h- | this study |
| 7516 | *bun107*-GFP::kanR *bun62::ura4+* *ade6*-M210 *ura4*-D18 *leu1*-32 h- | this study |
| 7570 | *bun62*-linker GFP::kanR *ade6*-M210 *ura4*-D18 *leu1*-32 h- | this study |
| 7868 | *sst2*-GFP::kanR *ade6*-M210 *ura4*-D18 *leu1*-32 h- | this study |
| 7869 | *sst2*-TAP::kanR *ade6*-M210 *ura4*-D18 *leu1*-32 h- | this study |
| 7875 | *bun107*-GFP::kanR *bun62*-TAP *ade6*-M210 *ura4*-D18 *leu1*-32 h- | this study |
| 7877 | *bun62*-linker GFP::kanR *bun107::ura4+ ade6*-M210 *ura4*-D18 *leu1*-32 h+ | this study |
| 7878 | *bun62*-linker GFP::kanR *ubp9::ura4+* *ade6*-M21X *ura4*-D18 *leu1*-32 h+ | this study |
| 7879 | *ubp9*-GFP::kanR *bun107::ura4+ bun62::ura4+* *ade6*-M210 *ura4*-D18 *leu1*-32 h+ | this study |
| 7882 | *bun107*-GFP::kanR *bun62*-TAP::kanR *ubp9::ura4+ ade6*-M21X *ura4*-D18 *leu1*-32 h+ | this study |
| 7903 | *nog1-*mCherry*::kanR ade6*-M210 *ura4*-D18 *leu1*-32 h+ | laboratory stock |
| 7983 | *ubp9*-TAP::kanR *bun107::ura4+* *ade6*-M210 *ura4*-D18 *leu1*-32 h+ | this study |
| 8088 | *bun62*-linker GFP *ubp9::ura4+ bun107::ura4+ ade6*-M21X *ura4*-D18 *leu1*-32 h- | this study |
| 8089 | *bun107*-GFP *ubp9::ura4+ bun62::ura4+* *ade6*-M21X *ura4*-D18 *leu1*-32 h+ | this study |
| 8316 | *bun62*-V5::kanR *ade6*-M210 *ura4*-D18 *leu1*-32 h+ | this study |
| 8317 | *bun107*-V5::kanR *ade6*-M210 *ura4*-D18 *leu1*-32 h+ | this study |
| 8319 | *rpn11*-TAP::kanR *ade6*-M210 *ura4*-D18 *leu1*-32 h- | this study |
| 8321 | *otu1*-TAP::kanR *ade6*-M210 *ura4*-D18 *leu1*-32 h- | this study |
| 8328 | *ubp9*-TAP::kanR *bun107::ura4+ bun62::ura4+* *ade6*-M210 *ura4*-D18 *leu1*-32 h- | this study |
| 8519 | *ubp9*-TAP::kanR *bun62::ura4+* *ade6*-M210 *ura4*-D18 *leu1*-32 h- | this study |
| 8521 | *ubp9*-GFP::kanR *bun107*-V5::kanR *bun62::ura4+* *ade6*-M210 *ura4*-D18 *leu1*-32 h- | this study |
| 8523 | *wsp1*::kanMX6 his3-D1 *ade6*-M216 *ura4*-D18 *leu1*-32 h- | V. Sirotkin |
| 8526 | *ubp9::ura4+* *myo1::kanR* *leu1-*32 *ade6*-M21x *ura4*-D18 h- | this study |
| 8528 | *ubp9::ura4+* *wsp1::kanMX6* *ade6*-M21X *ura4*-D18 *leu1*-32 h+ | this study |
| 8534 | *bun62*-V5::kanR *ubp9*-GFP::kanR *ade6*-M210 *ura4*-D18 *leu1*-32 h- | this study |
| 8535 | *bun107*-V5::kanR *ubp9*-GFP::kanR *ade6*-M210 *ura4*-D18 *leu1*-32 h- | this study |
| 8537 | *bun62*-V5::kanR *ubp9*-GFP::kanR *bun107::ura4+* *ade6*-M210 *ura4*-D18 *leu1*-32 h- | this study |
| 9156 | *sla2*::kanR *ade6*-M210 *ura4*-D18 *leu1*-32 h- | S. Castagnetti |
| 9158 | *ubp9::ura4+* *sla2::kanR* *ade6*-M21X *ura4*-D18 *leu1*-32 h- | this study |
| 9162 | *otu2*-linker TAP::kanR *ade6*-M210 *ura4*-D18 *leu1*-32 h- | this study |
| 9163 | *ubp1*-linker TAP::kanR *ade6*-M210 *ura4*-D18 *leu1*-32 h- | this study |
| 9165 | *ubp11*-linker TAP::kanR *ade6*-M210 *ura4*-D18 *leu1*-32 h- | this study |
| 9166 | *sfp47*-GFP::kanR *ade6*-M210 *ura4*-D18 *leu1*-32 h+ | this study |
| 9168 | *sfp47*-flag::kanR *ade6*-M210 *ura4*-D18 *leu1*-32 h+ | this study |
| 9172 | *ftp105*-V5::kanR *ade6*-M210 *ura4*-D18 *leu1*-32 h+ | this study |
| 9173 | *ubp5*-GFP::kanR *ftp105*-V5::kanR *ade6*-M210 *ura4*-D18 *leu1*-32 h+ | this study |
| 9175 | *ftp105::ura4+ ade6*-M210 *ura4*-D18 *leu1*-32 h+ | this study |
| 9181 | *ftp10*5-linker TAP::kanR *ade6*-M210 *ura4*-D18 *leu1*-32 h- | this study |
| 9184 | *rpn11*-GFP::kanR *ade6*-M210 *ura4*-D18 *leu1*-32 h- | this study |
| 9188 | *ubp4*-GFP::kanR *sfp47*-flag::kanR *ade6*-M210 *ura4*-D18 *leu1*-32 h- | this study |
| 9189 | *ftp105*-GFP::kanR *ura4*-D18 *leu1-32* h- | this study |
| 9190 | *ubp5::ura4+* *ura4*-D18 *leu1*-32 h- | this study |
| 9192 | *ubp5*-GFP::kanR *ftp105::ura4+* *ade6*-M210 *ura4*-D18 *leu1*-32 h+ | this study |
| 9194 | *ubp16*-linker TAP::kanR *ura4*-D18 *leu1*-32 h- | this study |
| 9306 | *ubp1*-linker GFP::kanR *ura4*-D18 *leu1*-32 h- | this study |
| 9307 | *ubp2*-linker GFP::kanR *ura4*-D18 *leu1*-32 h- | this study |
| 9308 | *ubp3*-linker GFP::kanR *ura4*-D18 *leu1*-32 h- | this study |
| 9309 | *ubp7*-linker GFP::kanR *ura4*-D18 *leu1*-32 h- | this study |
| 9310 | *ubp11*-linker GFP::kanR *ura4*-D18 *leu1*-32 h- | this study |
| 9311 | *ubp16*-linker GFP::kanR *ura4*-D18 *leu1*-32 h- | this study |
| 9312 | *uch2*-linker GFP::kanR *ura4*-D18 *leu1*-32 h- | this study |
| 9313 | *otu1*-linker GFP::kanR *ura4*-D18 *leu1*-32 h- | this study |
| 9314 | *otu2*-linker GFP::kanR *ura4*-D18 *leu1*-32 h- | this study |
| 9315 | *vrg4*-linker mCherry::ura4+ *ade6*-M216 *ura4*-D18 *leu1*-32 h- | S. Oliferenko |
| 9347 | *ubp5*-TAP::kanR *ftp105::ura4+* *ade6*-M210 *ura4*-D18 *leu1*-32 h+ | this study |
| 9353 | *sst2::ura4+ ubp4_156-593_::ura4+* *ubp5::ura4+ leu1-32 uraD18 h+* | this study |
| 9355 | *ubp4_156-593_::ura4+* *ura4-D18 leu1-32 h-* | this study |
| 9358 | *ubp4-GFP::kanR sfp47::ura4+ ade6-M210 ura4-D18 leu1-32 h-* | this study |
| 9359 | *ubp4-TAP::kanR sfp47::ura4+ ade6-M210 ura4-D18 leu1-32 h-* | this study |
| 9360 | *ftp105-GFP::kanR ubp5::ura4+ ura4-D18 leu1-32 h+* | this study |
| 9361 | *sfp47-GFP::kanR ubp4156-593::ura4+ ade6-M210 ura4-D18 leu1-32 h-* | this study |
| 9392 | *sfp47::ura4+ ura4-D18 leu1-32 h-* | this study |
| 9393 | *ubp4_156-593_::ura4+* *ubp5::ura4+ ubp9::ura4+ ura4-D18 leu1-32 h+* | this study |
| 9394 | *ubp4_156-593_::ura4+* *ubp5::ura4+ ubp9::ura4+ sst2::ura4 ura4-D18 leu1-32 h+* | this study |
| 9494 | *nog1-mCherry::kanR ubp16-linker GFP::kanR ade6-M210 ura4-D18 leu1-32 h+* | this study |
| 9606 | *ubp5-GFP::kanR vrg4-linker mCherry::ura4+ ade6-M21X ura4-D18 leu1-32 h+* | this study |
| 9615 | *ubp9::ubp9 (C50S)-TAP/kanR ade6-M21x ura4-D18 leu1-32 h+* | this study |
| 9616 | *ubp3-TAP::kanR ura4-D18 leu1-32 h-* | this study |
| 9617 | *ubp6-TAP::kanR ura4-D18 leu1-32 h-* | this study |
| 9618 | *ubp15-TAP::kanR ura4-D18 leu1-32 h-* | this study |
| 9619 | *ubp12-TAP::kanR ura4-D18 leu1-32 h-* | this study |
| 9622 | *ubp15::ura4+ ura4-D18 leu1-32 h-* | N. Kaüfer |
| 9706 | *ftp105-GFP::kanR vrg4-linker mCherry::ura4+ ura4-D18 leu1-32 h-* | this study |
| 10184 | *can1-HBH::kanR ade6-M210 ura4-D18 leu1-32 h-* | this study |
| 10189 | *ubp4_156-593_::ura4+ ubp5::ura4+ ubp9::ura4+ ubp15::ura4+ sst2::ura4+ leu1-32 ura4-D18 h+* | this study |
| 10190 | *ubp4_156-593_::ura4+ ubp9::ura4+ ubp15::ura4+ sst2::ura4+ leu1-32 ura4-D18 h+* | this study |
| 10191 | *ubp4_156-593_::ura4+ ubp5::ura4+ ubp9::ura4+ ubp15::ura4+ leu1-32 ura4-D18 h+* | this study |
| 10192 | *ubp4_156-593_::ura4+ ubp5::ura4+ ubp15::ura4+ sst2::ura4+ leu1-32 ura4-D18 h+* | this study |
| 10193 | *ubp5::ura4+ ubp9::ura4+ ubp15::ura4+ sst2::ura4+ leu1-32 ura4-D18 h-* | this study |
| 10194 | *ubp4_156-593_::ura4+ ubp5::ura4+ ubp15::ura4+ leu1-32 ura4-D18 h-* | this study |
| 10195 | *ubp5::ura4+ ubp9::ura4+ ubp15::ura4+ leu1-32 ura4-D18 h+* | this study |
| 10196 | *ubp5::ura4+ ubp15::ura4+ sst2::ura4+ leu1-32 ura4-D18 h-* | this study |
| 10197 | *ubp4_156-593_::ura4+ ubp9::ura4+ ubp15::ura4+ leu1-32 ura4-D18 h-* | this study |
| 10198 | *ubp4_156-593_::ura4+ ubp15::ura4+ sst2::ura4+ leu1-32 ura4-D18 h-* | this study |
| 10199 | *ubp9::ura4+ ubp15::ura4+ sst2::ura4+ leu1-32 ura4-D18 h-* | this study |
| 10200 | *ubp4_156-593_::ura4+ ubp9::ura4+ sst2::ura4+ leu1-32 ura4-D18 h-* | this study |
| 10201 | *ubp5::ura4+ ubp9::ura4+ sst2::ura4+ leu1-32 ura4-D18 h+* | this study |
| 10313 | *ost1-mCherry::ura4+ ade6-216 ura4-D18 leu1-32 h+* | S. Oliferenko |
| 10335 | *pub1-HBH kanR h+* | this study |
| 10404 | *ubp4-gfp/kanR pan1-mcherry/kanR ura4-D18 h+* | this study |
| 10457 | *ubp4::ubp4 (C236S-TAP)/kanR ura4-D18 leu1-32 h-* | this study |
| 10459 | *ubp5::ubp5 (C222S-TAP)/kanR ura4-D18 leu1-32 h-* | this study |
| 10477 | *pub1-HBH/kanR ubp4::ura4+ ubp5::ura4+ ubp9::ura4+ ubp15::ura4+ sst2::ura4+ leu1-32 ura4-D18 h+* | this study |
| 10478 | *can1-HBH/kanR ubp4::ura4+ ubp5::ura4+ ubp9::ura4+ ubp15::ura4+ sst2::ura4+ leu1-32 ura4-D18 h+* | this study |
| 10556 | *ubp15-mCherry::kanR pob1-GFP::kanR leu1-32 h+* | this study |
| 10807 | *tom70-GFP/kanR ade6-210 ura4-D18 leu1-32 h-* | this study |
